# A multilevel, multicomponent childhood obesity prevention group-randomized controlled trial improves healthier food purchasing and reduces sweet-snack consumption among low-income African-American youth

**DOI:** 10.1186/s12937-018-0406-2

**Published:** 2018-10-29

**Authors:** Angela C. B. Trude, Pamela J. Surkan, Lawrence J. Cheskin, Joel Gittelsohn

**Affiliations:** 1 Department of International Health, Global Obesity Prevention Center, and Center for Human Nutrition, Johns Hopkins Bloomberg School of Public Health, 615 N. Wolfe Street, Baltimore, MD 21205 USA; 2 Department of International Health, Social and Behavioral Interventions Program, Johns Hopkins Bloomberg School of Public Health, 615 N. Wolfe Street, Baltimore, MD 21205 USA; 3 Department of Health Behavior and Society, and the Global Obesity Prevention Center, Johns Hopkins Bloomberg School of Public Health, 550 N. Broadway, Baltimore, MD 21205 USA

**Keywords:** Consumption of sweets, Adolescent, Environmental intervention, African-American, Dietary intake, Childhood obesity

## Abstract

**Background:**

Consumption of foods and beverages rich in sugar remains high across all races and ages in the United States. Interventions to address childhood obesity and decrease sugar intake are needed, particularly in low-income settings.

**Methods:**

*B’more Healthy Communities for Kids* (BHCK) was a group-randomized, controlled trial implemented among 9–15-year olds in 30 low-income areas of Baltimore. We increased access to low-sugar foods and beverages at wholesalers and small food stores. Concurrently, we encouraged their purchase and consumption by children through youth-led nutrition education in recreation centers, in-store promotions, text messaging and a social media program directed at caregivers. Sugar consumption (sugar sweetened beverage (SSB), sweets) in youth was assessed pre- (*n* = 534) and post-intervention (*n* = 401) using the Block Kids Food Frequency Questionnaire. Purchasing of 38 healthier and 28 less healthier food/beverage varieties in the previous 7 days was assessed via self-report. Multilevel models at the community and individual levels were used. Analyses were stratified by age (younger: 9–12-year olds (*n* = 339) vs older: 13–15 (*n* = 170)). Models were controlled for child’s sex, race, total daily caloric intake, and caregiver’s age and sex.

**Results:**

Overall baseline mean healthier food purchasing was 2.5 (+ 3.6; min. 0, max. 34 items per week), and unhealthier food purchasing 4.6 (+ 3.7; 0–19 items per week). Mean intake at baseline for kcal from SSB was 176 (+ 189.1) and 153 (+ 142.5), and % of calories from sweets (i.e. cookies, cakes, pies, donuts, candy, ice cream, sweetened cereals, and chocolate beverages) was 15.9 (+ 9.7) and 15.9 (+ 7.7) in comparison and intervention youth, respectively. Intervention youth increased healthier foods and beverages purchases by 1.4 more items per week than comparison youth (β = 1.4; 95% CI: 0.1; 2.8). After the intervention, there was a 3.5% decrease in kcal from sweets for older intervention youth, compared to the control group (β = − 3.5; 95% CI: -7.76; − 0.05). No impact was seen on SSB consumption.

**Conclusion:**

BHCK successfully increased healthier food purchasing variety in youth, and decreased % calories from sweet snacks in older youth. Multilevel, multicomponent environmental childhood obesity programs are a promising strategy to improve eating behaviors among low-income urban youth.

**Trial registration:**

NCT02181010 (July 2, 2014, retrospectively registered).

**Electronic supplementary material:**

The online version of this article (10.1186/s12937-018-0406-2) contains supplementary material, which is available to authorized users.

## Introduction

The diet of youths today, especially in low-income, underserved urban populations, is high in refined carbohydrates, added sugar, fats, and salt [1]. Sugar intake is an important risk factor for diet-related chronic diseases, such as overweight and obesity [2], type-2 diabetes [3], and poor dental health [4].

A recent meta-analysis of cohort studies in children reported a significantly increased risk of being overweight or obese with consumption of one or more daily servings of sugar-sweetened beverages (SSBs) [2]. Although SSB consumption has declined slightly over the past decade, intake remains high, especially in youth, representing 10–15% of total caloric intake [5]. Importantly, African-American and Hispanic youth had greater increases in calories from sugar per capita than their white counterparts over the past three decades [6]. In addition, a recent nationally-representative study reported no decline (at 14% of total daily energy (calorie) intake) in the percentage of the total daily caloric intake from added sugar in U.S. children in the past decade, when considering both foods and beverages intake [7]. These findings suggest that foods such as grain-based desserts, candy, and other sweet snacks are important contributors of added sugar in children’s diet [8]. Children with higher intake of sugary beverages tend to snack more often than those with lower [9]. Furthermore, snacking patterns have changed in the past decade, as low-income children increased purchase and consumption of foods high in sugar [10], and increased consumption of foods away from home [11]. Snacks may significantly contribute to daily caloric intake, surpassing 27% of total daily calories among U.S. children aged 2–18 [9].

Dietary patterns are strongly influenced by a person’s food environment [12]. Food marketing and advertisements of unhealthier foods disproportionally target low-income minority populations [13]. Previous studies suggest that living in low-income areas where access to healthy food is limited increases risk of poor diets and obesity [14, 15]. Low-income individuals tend to live closer to small food stores with less availability of healthier foods and greater access to high-energy density food of low nutritional value, and increased portion sizes [16]. In Baltimore, low-income African-American youth reported visiting small food stores on average twice a day and buying chips, candy, and soda 2.5, 1.8, and 1.4 times per week, respectively [17]. In Philadelphia, 42% of low-income school-aged children shopped at corner stores twice a day, purchasing 350 calories each visit in candy, chips and SSBs [18].

Given the patterns in access to and marketing of unhealthier foods in low-income urban areas and the negative health outcomes associated with their consumption, there is a need to improve the community food environment. Due to the complex nature of eating behavior, solutions at the different levels of the socio ecological model (i.e., multilevel) paired with multiple actions within the same level (i.e., multicomponent) provide a promising population-based approach to leverage the food systems to promote health. Community-based intervention trials aiming at changing the food environment in and around the individual by improving availability and affordability of healthier foods at the community-level and concurrently improving demand and health literacy at the individual-level may be effective in reducing intake of high-sugar, high-fat beverages and snacks**.** The Shape Up Somerville (SUS) is one example of community-based multilevel multicomponent obesity prevention program that partnered with schools, restaurants and farmers’ market to improve availability of healthy menu options [19], and successfully increased availability of healthy foods in schools’ food service [20]. SUS found a statistically significant decrease in Body Mass Index (BMI) z-scores in children [21], and reduction in SSB intake after 2 years of intervention [22].

However, most community-based interventions have promoted healthier food alternatives at food stores targeting adults [23], and most multilevel interventions have been primarily school-based, targeted elementary- and middle-school -aged children [24], and few have demonstrated an impact on sugar intake or measured purchasing behaviors [25]. Thus, there is a need for community-based intervention trials to test strategies that target older children and adolescents [24]. Furthermore, previous longitudinal studies have reported important differences in food patterns across youth ages, with older youth (> 12 years old) snacking and purchasing foods out of the home more frequently than younger youth [26]. Therefore, it is important to investigate impact of nutrition interventions at different ages due to different food behaviors and societal eating norms, increased caloric intake, and changes in body composition.

The *B’more Healthy Communities for Kids* (BHCK) intervention was a multilevel, multicomponent childhood obesity prevention trial in Baltimore City that sought to modify the food environment outside of school [27]. Components of the intervention aimed at improving availability of healthier alternatives to high-sugar, high-fat beverages and snacks in small food stores and at increasing demand for these items through youth-led nutrition education sessions at community recreation centers to impact purchasing and consumption of healthier foods in youth (9–15 years old). This research addresses the following questions:

1. What was the impact of the multilevel BHCK intervention on purchasing behavior of healthier and unhealthier food items among youth?
2. What was the impact of the intervention among youth on the consumption of high-sugar, high-fat snacks and beverages?
3. How did the impact of the intervention differ between younger (9–12 years old) and older youth (13–15 years old)?

## Methods

### Study design

**Fig. 1:**
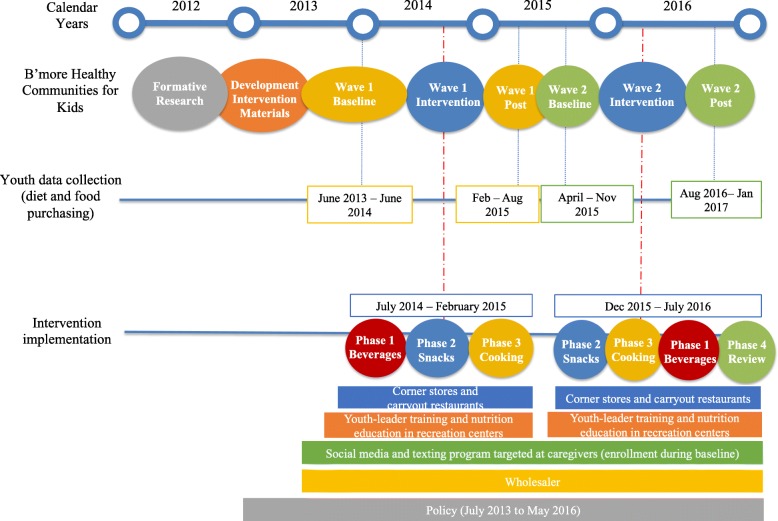
Overview of the timing of B’more Healthy Communities for Kids implementation and data collection

The BHCK intervention was a five-year funded multilevel, multicomponent childhood obesity prevention trial in Baltimore [27]. The intervention employed a group-randomized controlled trial design implemented at multiple levels of the urban food environment (policy, wholesalers, corner stores, carryout restaurants, recreation centers, and social media) to improve healthier food access, purchase, and consumption among low-income youth aged 9–15 and their caregivers living in food deserts in Baltimore City. The study used pre- and post-intervention assessment design, with two groups – intervention and comparison – implemented in two waves (wave 1: July 2014–February 2015; wave 2: December 2015–July 2016) (Fig. 1).

### Community randomization

BHCK took place in 30 zones, randomized to intervention (*n* = 7 in wave 1; n = 7 in wave 2) and comparison groups (n = 7 in wave 1; *n* = 9 in wave 2), using simple randomization. Assignment occurred publicly by drawing names of eligible recreation centers from a hat. A recreation center was at the nucleus of each zone, and zone’s eligibility criteria were: 1) predominantly African-American (> 50%); 2) low-income neighborhood (> 20% of residents living below the poverty line); 3) minimum of 5 small (< 3 aisles, no seating) food sources; 4) recreation center more than ½ mile away from a supermarket, and located in a food desert [28].

### Recruitment

**Fig. 2:**
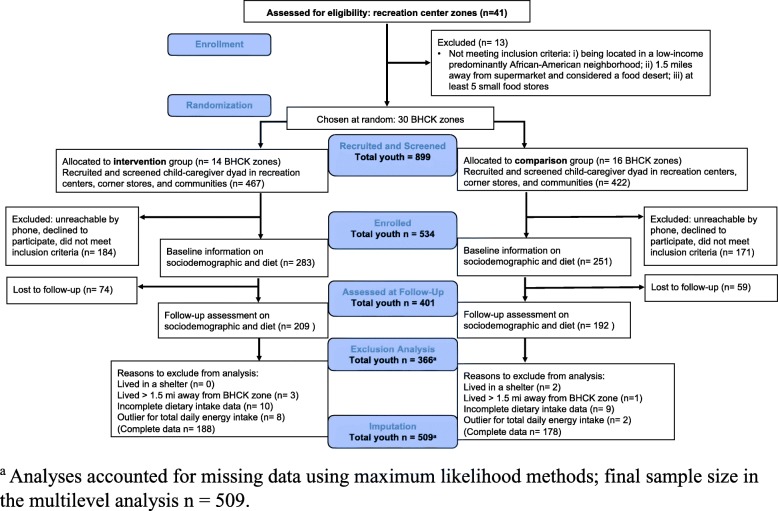
CONSORT flowchart of the randomization and course of the B’more Healthy Communities for Kids intervention

A sample of adult caregiver and child dyads were recruited at each recreation center and nearby corner stores in the 1.5-mile BHCK buffer zone. In each buffer zone, BHCK research assistants approached children and their caregivers about the study, and interested individuals provide their names and phone numbers. A list of 75–100 names per zone was entered into a sampling frame, with the goal to randomly select 20 dyads from each zone. Household eligibility criteria included: 1) at least one child in aged 9–15 years; 2) living in the same location for at least 1 month; and 3) not anticipating a move in the next 2 years [27]. An overview of study enrolment and participant flow is provided [see Fig. 2 and Additional file 1]. Groups assignments were concealed from the BHCK research assistants, who conducted the follow-up assessments.

### Promotion of healthier alternatives to beverages and snacks in the BHCK intervention

The BHCK intervention was divided into three phases, each lasting 2 months: 1) healthier beverages, 2) healthier snacks, and 3) healthier cooking methods. A fourth phase (review) was implemented in wave 2 only. During the healthier beverages phase, the program promoted healthier alternatives to SSBs [i.e., lower-sugar fruit drinks (25–75% less sugar than the original version), sugar-free drink mixes, zero-calorie flavored water, diet or low-sugar soda, and water] as part of each component (social media, small food stores, recreation center, youth-leader, wholesaler, policy) across all levels (individual, household, environmental, and policy). During the healthier snacks phase, BHCK promoted low-fat and low-sugar alternatives to unhealthier snacks, including low-fat yogurt, low-fat popcorn, fresh fruits, fresh vegetables, low-sugar granola bars, and mixed fruit in 100% fruit juice. In the healthier cooking phase, the intervention promoted cooking ingredients, such as low-sugar cereals, low-fat milk, 100% whole wheat bread, fresh/canned/frozen vegetables across all BHCK components.

### Multilevel multicomponent B’more healthy communities for kids intervention

BHCK encompassed 4 different socioecological levels – policy, environmental, interpersonal and intrapersonal – as well as multiple components involving wholesalers, small food stores (corner stores and carryout restaurants), recreation centers/peer-mentors, and social media. The BHCK components are described below.

#### Wholesaler

We partnered with three wholesalers in Baltimore City, and each was encouraged to stock BHCK-promoted food items. Foods and beverages were promoted through signage, in which a shelf-label was placed by a BHCK-interventionist in the wholesale stores highlighting the promoted item to storeowners [29]. Wholesalers also provided a $50 gift card to small stores participating in the program at the beginning of each phase to encourage initial stocking of a new promoted item (funded by BHCK).

#### Corner stores and carryout restaurants

We recruited 3–4 corner stores and carryout restaurants in each BHCK zone. We worked with storeowners in intervention zones to improve supply and demand for healthier options of food and beverages [29]. Small retailers were provided with gift cards from wholesalers, a stocking sheet with the promoted items, and were encouraged to stock at least one new promoted item every other week. Moreover, storeowners watched six training videos that provided information about the program, how to best improve customer relations, and use of healthier cooking methods (carryout owners only) [30, 31]. After completing each training module, owners were offered store supplies as a reward, ranging from produce baskets to refrigerators. To increase demand for healthier alternatives, we used materials and incentives (point-of-purchasing promotion and giveaways), and in-store taste tests, for example, fruit flavored water, baby carrots, and low-sugar granola bars, during two-hour educational sessions (delivered every other week in each intervention store by BHCK-interventionists). Posters and handouts promoting the food items were placed in all intervention stores.

#### Recreation centers

Prior to the intervention, youth leaders (Baltimore City college students) were trained by BHCK-interventionists in leadership and nutrition to conduct educational sessions in 14-intervention recreation centers with children through a peer-intervention approach [32]. Youth leaders were involved in the delivery of the intervention based on the perspectives of social cognitive theory, as a way to enable mentees to model mentors’ health behavior [33]. Fourteen sessions implemented every other week (total of 6 months) by youth leaders followed the themes of each BHCK phase. Nutrition sessions lasted 1 h, during which youth leaders implemented the BHCK nutrition curriculum with hands-on activities related to the different sugar and fat content in each drink and snack, and introduced a traffic light labeling method for beverages and snacks [34]. Giveaways and taste-tests were also conducted at the end of each session that aligned with the lesson. All children in the 9–15-year range attending the after-school program at the time of the intervention could participate in the nutrition education sessions. Although recruitment also occurred in recreation centers, study participant youth were not required to attend intervention sessions.

#### Social media and texting program

Social media (Facebook, Instagram, and Twitter) was used to integrate all levels of BHCK and targeted caregivers [35]. Recipes, news, and BHCK-specific activities related to healthier beverages and snacks were featured daily. The text message platform targeted mainly the caregiver level with goal setting strategies and BHCK educational activities for the specific BHCK zone. During each phase, caregivers received a text message 3 to 5 times a week related to healthier eating behavior. A text message example was: “*Water is much better than soda, but it doesn’t have 2 be boring. Try squeezing lemons or lime to add natural flavor & help u refresh this 4th of July weekend*”.

#### Policy

We worked with key city stakeholders to support policies for a healthier food environment in Baltimore, and to sustain BHCK activities. In addition, BHCK provided evidence-based information to support the development of policies at the city level using Geographic Information System (GIS)/System Science simulation model to simulate impact to aid stakeholder decision-making (e.g. urban farm tax credit, mobile meals, SSB warning labels) [36]. Implementation of the policy component is described in detail elsewhere [37].

Study participants were those interviewed at baseline/post-intervention, were not required to participate in any educational session (i.e., corner stores, carryout restaurants, and recreation centers), and only invited to follow social media and enroll in the texting program after their baseline appointments if in the intervention group. In-store and recreation center nutrition sessions were open to the public and delivered to anyone who was present at the time the intervention was delivered. In the comparison zones, neither recreation centers nor small food stores received the nutrition education sessions or communication materials, and caregiver-child dyads living in these areas were not enrolled in the BHCK text-messaging program. Hence, study participants were a potential sub-sample of the total population exposed to the BHCK intervention. By design, we believed that BHCK would reach its intended population by intervening in multiple settings that are key components of the community food environment. An additional pdf file shows examples of intervention materials [see Additional file 2] and a template for intervention description and replication (TIDieR) checklist is provided in the Additional file 3.

### Selection of BHCK promoted snacks and beverages

Promoted beverages and snacks qualified as *healthier* in the BHCK intervention were selected based on formative research and focus group discussions held with youth within the targeted age group [38]. These healthier alternatives were selected to be comparable in both flavor profile and price point to snack foods youth would normally purchase and consume. *Healthier* snacks and beverages in our study contained no more than 10% of the daily reference value for fat (i.e., below 6.5 g of fat per serving), 10 g of sugar per serving, and/or were good sources of fiber. These included low-fat string cheese, low-fat yogurt, low-sugar granola bars, fresh fruit, fruit cups in 100% juice, applesauce, sliced apples, popcorn, pretzels, baked chips, water, and low-sugar beverages. *Unhealthier* foods were snacks and beverages low in fiber and high in sugar and fat (i.e., above 6.5 g of fat and/or 10 g of sugar per serving), including baked goods, chocolate and non-chocolate candy, crackers, snack chips, soda, fruit punch, and sweetened tea.

### Training of interventionists and data collectors

BHCK-interventionists were graduate students, youth-leaders, public health educators, or dietitians trained in nutrition and health education. Data collectors, graduate students and staff, were trained intensively, including through role play and observation. They were masked after assignment to intervention. Data collectors gathered informed assent and consent from both the youth and caregiver, respectively. Following the interviews, data were checked for errors by the interviewer and a second research assistant. The data manager ensured that questionnaires had no missing pages or implausible values.

### Measures

#### Youth data collection

Baseline data were collected from June 2013 to June 2014 (wave 1) in a total of 299 youth and 298 caregivers, and from April to November 2015 (wave 2) in 235 caregiver-youth dyads [27]. Post-evaluation was conducted from March 2015 to March 2016 (wave 1) and from August 2016 to January 2017 (wave 2). Youth and caregivers received gift cards after each of the two interviews.

We did not analyze participants who had missing information for at least one outcome variable at baseline (*n* = 19), reported living in unstable housing arrangements such as in shelters or transitional housing (*n* = 2), lived more than 1.5 miles away from a BHCK recreation center (*n* = 4), or were considered an outlier for reported daily energy intake [39] (< 500 kcal/day or > 7000 kcal/day) (*n* = 10), yielding a total of 509 with complete baseline and 366 follow-up information for the analytical sample.

#### Youth purchasing behavior

Food purchasing behavior was assessed pre- and post-intervention (from 6 to 12 months after baseline). We used the Child Impact Questionnaire (CIQ) [40, 41] to collect food-related information in youth. The CIQ contained 79 questions pertaining to youth food purchasing habits, along with demographics [40–42]. The questionnaire was adapted on the basis of formative research from previous intervention trials in Baltimore [43, 44]. We pilot tested the questionnaire with youth (*n* = 20) for clarity and relevance of the instrument items.

We asked respondents to report what foods and beverages they obtained for themselves from different sources (e.g. corner stores, supermarkets, convenience stores, school, vending machines) in the 7 days prior. A list of 38 BHCK-promoted healthier foods and beverages and 28 unhealthier foods and beverages was provided. Information on construction of the food purchasing variable referring to the number of different items purchased per week of healthier and unhealthier foods are described in Table 1. Using methods similar to those previously published [45, 46], variety was defined as the total number of different food and beverage products (regardless of their sizes and flavors) assigning one point per purchase of each item in prior week.

**Table 1:** Food items purchased per week assessed in the Child Impact Questionnaire and construction of the food purchasing variety variables

Healthier foods items (*n* = 38)	1% or skim milk, diet soda, water, 100% fruit juice, sugar free drinks, fruit flavored water, unsweetened tea, fresh fruits such as apples, oranges, bananas, frozen and canned fruit, fresh, frozen, and canned vegetables, canned tuna in water, low sugar/high fiber cereals, 100% whole wheat bread, hot cereal, pretzels, baked chips, reduced-fat chips, dried fruit, nuts or seeds, cooking spray, grilled chicken, grilled seafood, fruit and vegetable as side dishes, deli sandwich, tacos, yogurt, granola	Healthier food purchasing variety (observed) ^a^ Maximum score: 34 Minimum score: 0 Mean: 2.6 Standard deviation: 3.6 Cronbach’s alpha: 0.87
Unhealthier foods items (*n* = 28)	whole milk, 2% milk, regular soda or regular energy drinks, fruit drinks, sweetened iced tea, sports drinks, applesauce, sugary cereals, white bread or split top wheat, burger, pizza, fried chicken, fried seafood, fries, fried chicken sandwich, carryout-Chinese food, chips, baked goods (cookies, cakes, poptarts), chocolate candy, ice cream, juice popsicles, snow cones, other candies.	Unhealthier food purchasing variety (observed) ^a^ Maximum score: 19 Minimum score: 0 Mean: 4.6 Standard deviation: 3.7 Cronbach’s alpha: 0.80

^a^For the construction of the food purchasing variable referring to the number of different items purchased per week, we first assigned one point to each food/beverage item the youth reported purchasing in the past 7 days, or 0 if they did not purchase that item. Then, we summed all the items belonging to “healthier foods” to derive the healthier food purchasing variety variable, and separately summed those under “unhealthier items” to derive the unhealthier food purchasing variety variable. Maximum, minimum, means, and standard deviations are reported based on the baseline number of different items purchased per week observed among children in BHCK. Underline text represents the name of the variable constructed

#### Youth food and beverage intake

The Block Kids 2004 Food Frequency Questionnaire (BKFFQ) instrument was used to collect sugar, fat, beverage, and snack intake in youth [47]. This is a semi-quantitative questionnaire, validated in adolescent populations [47, 48] that ascertains the previous week’s frequency (from ‘none’ to ‘every day’) and consumption amount of 77 common food items (with three to four categories related to food type). It contains foods identified by NHANES II commonly consumed by youth. Completed FFQs were analyzed by Nutrition Quest (Berkley, California, USA) for each youth.

Daily fruit and vegetable intake were estimated in cup-equivalent servings. Vegetable servings exclude potatoes and legumes, and fruit servings include 100% fruit juice. NutritionQuest also calculated the daily sugary beverage intake in kilocalories, and added sugars (sugars and syrups that are added to foods during processing or preparation) in teaspoon-equivalents. Percentage of kcal from sweets was calculated as total kcal coming from sweets and grain-based desserts (sweet cereal, ice cream, cookies, donuts, cake, chocolate candy, other candy, chocolate milk, pudding flan) divided by the total kcal from the whole diet as the denominator. The food groups for the BKFFQ database were developed using National Health and Nutrition Examination Survey (NHANES) and the USDA’s My Pyramid Equivalents Database 2.0 (MPED).

#### Covariates

Sociodemographic characteristics of youth and their caregivers were collected at baseline and post-evaluations using the Child Impact Questionnaire (CIQ) for youth’s age and sex, and the Adult Impact Questionnaire (AIQ) [49] for caregiver and household information. The AIQ included questions on demographics and household socioeconomics: caregiver’s age (continuous variable), sex (female, male), education level (categorized into < high school, completed high school, and > high school), household annual income (US$0–10,000; 10,001-20,000; 20,001–30,000 or higher), housing arrangement (owned, rent, and shared with family or other arrangement (group housing, transitional housing)), number of individuals in the household (continuous variable), and food assistance participation (received WIC (Special Supplemental Nutrition Program for Women, Infants, and Children) or SNAP (Supplemental Nutrition Assistance Program) benefits in the past year). Sociodemographic variables that were statistically different at a *p*-value < 0.05 between intervention and comparison groups were included in adjusted effect models as potential confounders.

#### Power calculation

The BHCK study was powered to detect a difference in healthy food purchasing score of 4–5 items per week, between intervention and control groups [27]. We drew upon our previous study baseline data on adult food purchasing to address the proposed hypothesis [50]. To estimate the sample size and the detectable difference estimate, an analysis was conducted prior to implementation of BHCK accounting for 30 recreation center zones (unit of randomization), controlling for a power of 80% (1**-**β) and a probability of a type I error of α **=** 0.05 (two-sided), and assuming a 20% drop-out after 2 years.

#### Data analysis

Statistical analysis was conducted using Stata 13.1 (College Station, TX, 2013). Means and standard deviations (SD) were estimated for key baseline descriptors. Continuous variables were tested for differences between intervention group and comparison group with independent 2-tailed t-test, and the Chi-square test for proportions was used for categorical variables.

The intervention effects on the mean change in diet and food-purchasing behaviors were assessed by the difference between the mean change of the outcome in the intervention compared to the control groups using a multilevel linear mixed-effect model. The multilevel model had mixed-effect components that accounted for both fixed and random effects. The single fixed independent variable included the time-by-group interaction. The random effect allowed recreation center zone’s coefficients and the random variation among repeated measures in the youth to vary randomly at the group level. The intraclass correlation coefficients (ICC) for the outcome measures at the subject-within-zone level ranged from 0.34–0.13. If the estimate was significant (*p* < 0.05), the null hypothesis (that mean dietary and food purchasing outcomes are equal in the intervention and control groups after the BHCK intervention) was rejected.

Caregiver’s age (continuous), and youth’s age (continuous, centered at the mean), caregiver and youth’s sex, and race were added as covariates in the food-purchasing models. In the dietary intake models, we included the following covariates: caregiver and youth’s age and sex, youth’s race, and total daily caloric intake [51]. We found statistically significant differences between youth who were retained in the intervention versus those lost to follow-up in terms of their caregiver’s age and sex. Missing data were imputed by modeling and estimating both the means and the random effect jointly using all non-missing data in the covariate matrix (maximum likelihood estimation) to address potential bias due to loss to follow-up and to maximize sample size (*n* = 509) [52]. Therefore, to control for selection bias, all models were controlled for variables that were predictors of dropout. Impact analyses were also stratified by age category: 9–12 and 13–15 year olds.

This study was approved by the Johns Hopkins Bloomberg School of Public Health Institutional Review Board (IRB #00004203).

## Results

Each component of the BHCK program was evaluated through detailed process evaluations reported elsewhere [29, 31, 34, 35, 37]. Overall, the environmental component (wholesaler, corner store, and carryout restaurants), policy, and the nutrition-education component (youth-led recreation center sessions) were implemented with moderate-to-high reach, dose delivered, and fidelity [53].

### Baseline characteristics of the BHCK youth sample

The vast majority of our study sample self-identified as African-American (overall average 96.6%), and 49% of youth were either overweight or obese (Table 2). Most youth were from a household that received SNAP (70.8%), with a female primary caregiver (93.2%). Significant differences were found between treatment groups with respect to youth age categories, with the proportion of older youth (13–15 years old) being somewhat higher in the intervention group (*p* = 0.03), and youth’s caregiver age (*p* = 0.02).

**Table 2:** BHCK low-income urban African-American youth’s socio-demographic characteristics at baseline

Baseline Characteristics	n (509)	Intervention	Comparison	*p*-value
		(*n* = 273)	(*n* = 236)	
Youth				
Gender				
Male (%)	227	45.9	42.8	0.45
Female (%)	282	54.1	57.2	
Age (years) - Mean (SD)		11.7 (1.3)	11.9 (1.6)	0.11
9–12 (%)	339	70.7	61.8	0.03^a^
13-15 (%)	170	29.3	38.1	
Race – African-American (%)	493	95.9	97.5	0.94
BMI (age- and sex-specific category)				
Normal weight (%)	260	48.9	55.3	0.20
Overweight (%)	117	23.9	22.1	
Obese (%)	127	27.2	22.6	
Total caloric intake (kcal) - Mean (SD)	509	1692.5 (915.4)	1777.2 (1107.9)	0.34
Caregiver				
Gender – Female (%)	508	92.3	90.7	0.5
Age (years) – Mean (SD)	506	38.5 (8.9)	40.3 (9.7)	0.02^a^
Education Level				
< High School (%)	89	19.5	15.3	0.5
High School (%)	204	39.3	41.3	
> High School (%)	214	41.2	43.4	
Household				
Individuals in the household - Mean (SD)	508	4.5 (1.6)	4.6 (1.6)	0.50
Annual Income (US$)				
0–10,000 (%)	120	25.7	21.2	0.16
10,001–20,000 (%)	116	19.1	27.1	
20,001–30,000 (%)	92	19.1	16.9	
> 30,000 (%)	180	36.0	34.7	
Food Assistance Participation				
SNAP (%)	372	75.4	70.7	0.30
WIC (%)	114	22.4	22.4	0.99
Housing Arrangement				
Living w/ family or other (%)	54	8.8	12.7	0.10
Rented (%)	344	68.7	66.5	
Owned (%)	110	22.4	20.7	

Abbreviation: *BMI* Body Mass Index, *SD* standard deviation, *SNAP* Supplemental Nutrition Assistance Program, *WIC* The Special Supplemental Nutrition Program for Women, Infants, and Children

^a^Intervention groups are statistically different when comparing the proportion of youth and caregiver characteristics using the chi-square test or two-tailed t-test

### Impact of BHCK intervention on purchase of healthier and unhealthier food by youth

We found a significant positive effect of the intervention on the variety of healthier food purchased among intervention youth versus comparison youth (Table 3). Overall, youth in the intervention group purchased 1.4 more healthier foods and beverages per week than comparison youth (β = 1.4; 95% CI: 0.1; 2.8), after controlling for caregivers’ age and sex, youths’ age, sex, and race.

**Table 3:** Adjusted differences in purchasing behaviors between intervention and comparison youth after BHCK intervention ^a,b^

Youth Purchasing Behavior	Predictive Baseline		Predictive Post-intervention		Pre-post change: adjusted difference ^c^
	Intervention	Comparison	Intervention	Comparison	
	Mean (SE)	Mean (SE)	Mean (SE)	Mean (SE)	Effect (95% CI)
Healthier Food					
Items per week	2.6 (0.9)	3.2 (0.9)	11.4 (0.9)	10.6 (0.9)	**1.4 (0.02; 2.8)**
9–12 years old	2.4 (0.9)	3.4 (0.9)	11.9 (0.9)	10.2 (0.9)	**2.8 (0.9; 4.6)**
13–15 years old	3.5 (1.0)	2.9 (1.0)	9.6 (1.1)	10.3 (1.0)	−1.4 (−3.6; 0.8)
Unhealthier Food					
Items per week	4.6 (0.6)	5.0 (0.6)	10.7 (0.6)	10.1(0.6)	0.9 (−0.2; 2.1)
9–12 years old	4.2 (0.6)	4.7 (0.6)	10.9 (0.6)	9.9 (0.6)	**1.6 (0.1; 3.0)**
13–15 years old	6.0 (0.8)	5.4 (0.7)	9.9 (0.9)	10.0 (0.8)	−0.7 (−2.6; 1.2)

Abbreviations: *SE* (standard error), *CI* (confidence interval)

^a^Multilevel models were conducted with Stata 13.1 package with the maximum likelihood option to impute multilevel data (n = 509). Multilevel models are good approach to be used under the missing at random assumption, as it models both the means and the random effect jointly [52]

^b^In all models: treatment group was coded as comparison (0) and intervention (1); time was coded as baseline (0) and post-intervention (1); caregiver’s age (continuous), and youth’s age (continuous, centered at the mean), caregiver and youth’s sex (0 = male, 1 = female), and race (0 = African-American, 1 = other) were added as covariates; standard errors were corrected for clustering for repeated measures from the same individual and BHCK neighborhood (from 1 to 30)

^c^Mean adjusted difference in change over time for intervention compared to control youth

Healthier food (low fat/low sugar) by variety of different number of food items purchased per week, includes: *1% or skim milk, diet soda, water, 100% fruit juice, sugar free drinks, fruit flavored water, unsweetened tea, fresh fruits such as apples, oranges, bananas, frozen and canned fruit, fresh, frozen, and canned vegetables, canned tuna in water, low sugar/high fiber cereals, 100% whole wheat bread, hot cereal, pretzels, baked chips, reduced-fat chips, dried fruit, nuts or seeds, cooking spray, grilled chicken, grilled seafood, fruit and vegetable as side dishes, deli sandwich, tacos, yogurt, granola*

Unhealthier food (high fat/high sugar) by variety of different number of food items purchased per week, includes*: regular soda, fruit punch, sweet ice tea, whole milk, tuna in oil, pork hot dog, baked beans, sugar cereal, white bread, sweetened oatmeal, chips, cookies, candy, ice cream, popsicle, butter, oil, mayonnaise*

**Bolded** values: p < 0.05

Youth between 9 and 12 years old at baseline in the intervention group purchased 2.8 greater healthier food items per week, and 1.6 more items per week of unhealthier foods over time, when compared to youth in the comparison group (change in number of different items per week of healthier food purchases: β = 2.8; 95% CI: 0.9; 4.6; items per week of unhealthier food purchases: β = 1.6; 95% CI: 0.1; 3.0). There was no impact on food purchasing behavior among the older youth in the stratified analysis.

### Impact of BHCK intervention on dietary intake in youth

We found a significant effect of the BHCK intervention on the decrease in % of kcal from sweet snacks and desserts (i.e. cookies, cakes, pies, donuts, candy, ice cream, sweetened cereals, and chocolate beverages) among older intervention youth (13–15 years) compared to older control youth (β = − 3.5; 95% CI: -7.0; − 0.1) (Table 4). We did not find a statistically significant change in SSBs (total kcal or daily ounces), and fruits and vegetables (daily serving) between intervention and control youth over time.

**Table 4:** Adjusted differences in consumption behaviors between intervention and comparison youth after BHCK intervention ^a,b^

Youth Daily Consumption	Predicted Baseline		Predicted Post-intervention		Pre-post change: adjusted difference ^c^
	Intervention	Comparison	Intervention	Comparison	
	Mean (SE)	Mean (SE)	Mean (SE)	Mean (SE)	Effect (95% CI)
Total daily caloric intake	1706.9 (65.5)	1771.3 (67.8)	1358.1 (73.4)	1349.9 (75.8)	72.5 (−120.3; 265.4)
9–12 years old	1712.1 (76.7)	1669.4 (84.2)	1360.5 (85.7)	1318.2 (92.5)	−0.5 (− 240.4; 239.5)
13–15 years old	1678.4 (104.3)	1927.7 (96.8)	1377.6 (104.3)	1437.9 (108.4)	189.1 (− 132.9; 510.9)
Beverage					
Sugary beverages (total kcal)	147.8 (8.1)	160.3 (8.6)	181.7 (9.6)	170.6 (10.1)	23.4 (−7.3; 54.1)
9–12 years old	125.0 (9.1)	138.7 (10.3)	180.1 (10.7)	168.0 (11.8)	25.8 (−10.1; 61.7)
13–15 years old	182.7 (15.9)	188.6 (15.1)	201.1 (19.4)	196.3 (17.1)	10.7 (−47.2; 68.7)
Fruit Punch (ounces, daily)	4.7 (0.5)	5.5 (0.5)	5.3 (0.4)	5.2 (0.4)	0.8 (−1.0; 2.5)
9–12 years old	3.9 (0.6)	4.5 (0.6)	5.2 (0.5)	4.8 (0.5)	0.9 (−1.2; 3.1)
13–15 years old	5.6 (0.8)	6.4 (0.9)	5.9 (0.9)	6.5 (0.8)	0.1 (−3.1; 3.3)
Snacks					
% of kcal from sweets	14.9 (0.6)	15.2 (0.6)	14.5 (1.9)	15.8 (0.7)	−1.0 (−3.1; 1.2)
9–12 years old	15.5 (0.6)	16.1 (0.7)	14.9 (0.7)	15.5 (0.8)	0.1 (−2.7; 2.8)
13–15 years old	15.1 (0.9)	14.3 (0.8)	14.7 (0.9)	11.9 (1.1)	**−3.5 (−7.0; −0.1)**
Dietary total sugar (grams)	120.3 (2.2)	117.2 (2.3)	121.1 (2.6)	115.7 (2.7)	2.3 (−6.5; 11.0)
9–12 years old	117.8 (2.5)	113.6 (2.8)	119.4 (2.9)	113.5 (3.2)	1.6 (−8.7; 11.9)
13–15 years old	125.7 (4.1)	124.6 (3.8)	123.3 (5.0)	119.3 (4.4)	2.9 (−13.1; 19.1)
Dietary sodium (mg)	2321.6 (28.0)	24,702.9(29.6)	2326.0 (33.3)	2415.7 (34.7)	−8.4 (− 117.6; 100.8)
9–12 years old	2259.9 (31.5)	2360.5 (36.1)	2281.5 (37.5)	2376.7 (41.3)	5.4 (− 127.9; 138.8)
13–15 years old	2446.1 (54.1)	2484.6 (50.9)	2427.9 (65.5)	2497.1 (57.5)	−30.5 (− 219.9; 158.3)
Fruit (total cups)	1.7 (0.1)	1.4 (0.1)	1.4 (0.1)	1.2 (0.1)	−0.1 (−0.3; 0.2)
9–12 years old	1.8 (0.1)	1.5 (0.1)	1.3 (0.1)	1.2 (0.1)	−0.1 (− 0.5; 0.1)
13–15 years old	1.5 (0.1)	1.5 (0.1)	1.4 (0.1)	1.1 (0.1)	0.2 (−0.1; 0.6)
Vegetable (total cups)	0.9 (0.1)	1.0 (0.1)	0.8 (0.1)	0.9 (0.1)	−0.1 (− 0.1; 0.1)
9–12 years old	1.0 (0.1)	1.1 (0.1)	0.8 (0.1)	0.9 (0.1)	−0.1 (− 0.2; 0.1)
13–15 years old	0.9 (0.1)	0.9 (0.1)	0.9 (0.1)	0.8 (0.1)	0.1 (−0.2; 0.2)
Fat (servings)	3.1 (0.1)	3.0 (0.1)	3.2 (0.2)	3.2 (0.1)	−0.1 (− 0.2; 0.2)
9–12 years old	3.3 (0.1)	3.2 (0.1)	3.2 (0.1)	3.2 (0.1)	−0.1 (− 0.5; 0.2)
13–15 years old	3.3 (0.1)	3.2 (0.1)	3.2 (0.1)	3.2 (0.1)	−0.1 (− 0.5; 0.2)

Abbreviations: *SE* (standard error), *CI* (confidence interval)

^a^Multilevel models were conducted with Stata 13.1 package with the maximum likelihood option to impute multilevel data (n = 509). Multilevel models are good approach to be used under the missing at random assumption, as it models both the means and the random effect jointly [52]

^b^In all models: treatment group was coded as comparison (0) and intervention (1); time was coded as baseline (0) and post-intervention (1); caregiver’s age (continuous), and youth’s age (continuous, centered at the mean), caregiver and youth’s sex (0 = male, 1 = female), race (0 = other; 1 = African-American), total daily caloric intake (continuous) were added as covariates; standard errors were corrected for clustering for repeated measures from the same individual and BHCK neighborhood (from 1 to 30)

^c^Mean adjusted difference in change over time for intervention compared to control youth

**Bolded** values: p < 0.05

## Discussion

To our knowledge, this is the first study to evaluate youth dietary behavior changes that resulted from a randomized, multilevel community food environment-based (non-school) obesity prevention trial in a low-income urban food desert setting. After the BHCK intervention, youth in the intervention group purchased almost 1.5 additional types of healthier food/beverage items per week, compared to their counterparts. This finding is supported by our other results, as BHCK was successful in improving availability of healthier foods and beverages in small food stores in intervention zones [54], indicating that food availability and promotion at the point-of-purchase may shape people’s food choices. Few community-based interventions have assessed the impact of the program on food purchasing behaviors in youth. For instance, a previous trial implemented in Baltimore with a similar study population did not find an impact of the intervention on food purchasing behavior among youth [45]. Researchers attributed the lack of intervention effect on their limited ability to make changes at the structural level. Conversely, the BHCK trial was one of the first studies to involve wholesalers to guarantee healthier food availability throughout the food supply chain [29]. Partnering with three wholesalers in the city was one of the innovative approaches used by BHCK to ensure that storeowners would be able to find and stock promoted foods and beverages, and may have led to greater, more sustained changes in the food environment.

The age-stratified analysis demonstrated that BHCK decreased kcal intake from sweet snacks among older youth in the intervention group by 3.5% compared to their counterparts. This is encouraging, given that sweet snacks are among the most frequently purchased items by youth in corner stores [17, 18]. Youth have almost doubled sweet snack intake in the past three decades, and it is among the main sources of added sugar intake in U.S. children [8, 55].

Another multilevel childhood obesity intervention trial that was implemented in low-income communities in Travis County, Texas (CATCH) found a 0.6 lower unhealthier food index consumption (i.e. fatty meats, fried meat with a crust, French fries/chips, white bread, fruit punch, sodas, frozen desserts, sweet rolls/cake, chocolate candy, and other candy) among middle-school aged children in the school-plus-community arm, compared to school-only intervention [56]. A four-year childhood obesity intervention with Swedish youth that restricted access to sweets and SSBs in the school food environment was also successful in decreasing sweets intake among youth and their families [57]. Compared to younger youth, older youth have an overall lower dietary quality [58], experience greater autonomy, and may be more influenced by the community food environment [59]. Therefore, efforts to improve the community food environment may be effective in changing dietary patterns in older youth and adolescents.

There was no significant intervention effect on fruit and vegetable consumption and SSB intake among youth. A possible explanation is that, although BHCK promoted fruit and vegetable intake at the store, recreation center, and social media/texting levels, fruits and vegetables were not the only promoted food items in the snacks and cooking phases. Furthermore, fruits and vegetables comprise only 1.0% of items purchased by low-income African-American children and adolescents in corner stores, as reported in a previous study conducted in Philadelphia, PA, U.S. [46]. However, other multilevel childhood obesity interventions have reported a positive impact on fruit and vegetable intake in youth [60–62], demonstrating that this may be an effective approach to improve youth’s diet quality. Nevertheless, future interventions should test different approaches to improve fruit and vegetable intake among low-income African-American youth, perhaps by focusing on the promotion of frozen, canned, and fresh produce, while decreasing consumption barriers such as price, food quality, and convenience. Given that African-American youth have lower dietary quality than other groups in the U.S., it is imperative that future interventions and policies focus on improving healthier food intake in this population [58].

Even though both healthier and unhealthier purchasing increased over time, total daily caloric intake declined from baseline to post-intervention evaluation among both groups. Although BHCK promoted low-sugar beverages and water during the beverage phase, the intervention did not restrict availability of these items in participating retail food store and in after-school programming refreshment stand/vending machines. In addition, it is possible that youth may have substituted high-fat, high-sugar beverages and snacks with lower-fat, lower-sugar options that were not captured by the BKFFQ, such as low-fat string cheese, low-sugar beverages, fruit cups in 100% juice, etc., which may help explain the decline in calorie intake observed over time. Food and beverage substitutions may also explain the lack of effect of BHCK on total daily caloric intake. Moreover, although SSB still represents more than 10% of total caloric intake, the percentage of total daily energy from SSB seems to be decreasing since 2000, and has reached a plateau among all youth ages and races in the U.S. [63]. Although the Shape Up Somerville intervention successfully decreased unhealthier intake in children (− 2.0 oz of SSB/day), they also did not find an effect of the intervention on children’s daily fruit and vegetable intake [22].

Interestingly, we found that the BHCK intervention increased the number of different items purchased per week of both healthier and unhealthier foods among children 9–12 years of age. Our finding suggests that although improving availability and promotion of healthier food was effective both overall and among younger youth, this age group did not decrease the number of different items of unhealthier food items purchased. Research with 10–12 year-old youth in New York found that substitution of healthier for unhealthier food is related to how much money a child has available [64], suggesting that low-income youth are more likely to change food purchasing patterns if unhealthier food prices are increased. Therefore, pricing strategies could be effective in improving healthier food purchasing in this population.

We also noted that healthier food purchasing increased substantially in both groups. A possible explanation is that all BHCK corner stores improved their Healthy Food Availability Index (HFAI) score, although the largest change was seen among intervention corner stores (mean change HFAI among comparison corner store: 1.67 versus intervention: 5.65, *p* = 0.01) [54]. As BHCK worked with three wholesalers, important food suppliers to all Baltimore corner stores, it is possible that other store owners not receiving the intervention were driven to stock more foods in their stores from the healthier group when exposed to newer healthier food items at the time they visited wholesalers [54]. Furthermore, the overall increase in number of healthier foods purchased among youth over time may reflect an overall gain in purchasing power as youth get older [46].

Another explanation for the positive effect on healthier food purchasing variety (overall and among younger youth) may be the nutrition education sessions conducted with youth in recreation centers and the in-store point-of-purchase promotions with repeated taste tests of the promoted healthier snacks. This hypothesis may be supported by the fact that younger youth were more likely to be exposed to the community nutrition education intervention than older youth [65]. Furthermore, it is possible that other social and household factors influenced youth to purchase fewer types of either healthier or unhealthier foods than the environmental factors accounted for in the BHCK intervention.

Limitations to this study should be noted. First, this study experienced a higher attrition rate than initially projected (24.9%), thus decreasing the final sample size, despite efforts to avoid drop-outs (e.g. eligibility criteria included intent to stay within the study areas over the next 2 years, multiple attempts to contact the families by phone, and using household visits to conduct follow-up surveys). However, when we compared baseline characteristics between individuals with completed follow-up evaluation and missing informants regarding covariates and outcomes, we identified that youth with a female and older caregiver were more likely to remain in the study. Thus, to address potential selection bias, we included caregiver’s age and sex as covariates in all multilevel regression models, and used maximum likelihood methods to produce unbiased estimates for data missing at random. Second, randomization at the individual-level was not possible. Nevertheless, selection bias was also ameliorated by having a comparison group of youth sampled from similar neighborhoods. Given that participating youth were low-income urban African Americans, results may not be generalizable to other populations. Third, due to the nature of the multilevel, multicomponent community-based trial, it was not possible to identify which specific components of the BHCK intervention led to changes in diet and food purchasing behaviors. In addition, although the program was implemented according to our initial process evaluation standards, achieving optimal intensity of the intervention (e.g. form of delivery, duration, and frequency) is challenging [66], and may partially explain the modest impact on dietary intake. Fourth, BHCK was implemented in two waves at different times (1 year apart). Although the structure of the intervention remained the same across the waves, improvements in the design of intervention materials and lessons learned from wave 1 were implemented during wave 2 (e.g.: increased size of posters, implemented ‘review phase’ to increase duration of the intervention, increased frequency of social media posts from weekly to daily). However, a sensitivity analysis with an additional interaction term to explore potential differences in impact by wave did not show any statistically significant differences between waves. Fifth, although multiple testing is a concern, we explored differences between only two categories of ages based on a priori hypothesis and distinct food-related behaviors previously reported in the literature. Lastly, to minimize respondent burden, we did not gather information on the quality or quantity of food acquired by the youth when collecting data on food purchased. Furthermore, in our food acquisition survey, we asked youth to only report on food acquisition when they were purchasing food for themselves (without including food that others purchased for them). We did not examine test-retest reliability of the youth food purchasing survey; however, information bias was minimized by the randomized design and the statistical methods employed. Future studies investigating food purchasing patterns in youth should explore changes in frequency, quantity, and amount of money spent on food.

## Conclusions

We found that intervening in the community food environment concomitantly with nutrition education in after-school settings may be a promising strategy to drive healthier food purchasing and decrease intake of sweet snacks among low-income, urban, African-American youth. Our findings support the effectiveness of a multilevel, multicomponent nutrition intervention program in improving healthier food purchasing behavior and decreasing caloric intake from less healthier foods, adding to evidence from previous studies. This study provides evidence-based information suggesting that intervening in the environment and improving healthier food access in food deserts can impact food behaviors among youth, which may lead to decreased prevalence of obesity and improved health outcomes. However, it is crucial that changes in healthier food access be supplemented by promotional activities to increase demand.

## Additional files

Additional file 1: CONSORT checklist of the B’more Health Communities for Kids program. (DOCX 262 kb)

Additional file 2: This file contains pictures of communication and intervention materials used during the implementation of the B’more Healthy Communities for Kids intervention. (PDF 22812 kb)

Additional file 3: TIDieR Checklist of the intervention activities of the B’more Healthy Communities for Kids. (DOCX 30 kb)

## Abbreviations

(AIQ): Adult Impact Questionnaire
(BHCK): B’more Healthy Communities for Kids
(BKFFQ): Block Kids 2004 Food Frequency Questionnaire
(BMI): Body Mass Index
(CIQ): Child Impact Questionnaire
(GIS): Geographic Information System
(HFAI): Healthy Food Availability Index
(NHANES): National Health and Nutrition Examination Survey
(SD): Standard deviations
(SNAP): Supplemental Nutrition Assistance Program
(SSB): Sugar Sweetened Beverages
(SUS): Shape Up Somerville
(WIC): Special Supplemental Nutrition Program for Women, Infants, and Children

## References

[1] Popkin BM, Adair LS, Ng SW (2012). Global nutrition transition and the pandemic of obesity in developing countries. Nutr Rev.

[2] Te Morenga L, Mallard S, Mann J (2013). Dietary sugars and body weight: systematic review and meta-analyses of randomised controlled trials and cohort studies. BMJ (Clinical research ed).

[3] Imamura Fumiaki, O'Connor Laura, Ye Zheng, Mursu Jaakko, Hayashino Yasuaki, Bhupathiraju Shilpa N, Forouhi Nita G (2016). Consumption of sugar sweetened beverages, artificially sweetened beverages, and fruit juice and incidence of type 2 diabetes: systematic review, meta-analysis, and estimation of population attributable fraction. British Journal of Sports Medicine.

[4] Bernabé E, Vehkalahti MM, Sheiham A, Aromaa A, Suominen AL (2014). Sugar-sweetened beverages and dental caries in adults: a 4-year prospective study. J Dent.

[5] Han E, Powell LM (2013). Consumption patterns of sugar-sweetened beverages in the United States. J Acad Nutr Diet.

[6] Wang Y, Beydoun MA, Liang L, Caballero B, Kumanyika SK (2008). Will all Americans become overweight or obese? Estimating the progression and cost of the US obesity epidemic. Obesity (Silver Spring, Md).

[7] Powell ES, Smith-Taillie LP, Popkin BM (2016). Added sugars intake across the distribution of US children and adult consumers: 1977-2012. J Acad Nutr Diet.

[8] Slining MM, Popkin BM (2013). Trends in intakes and sources of solid fats and added sugars among US children and adolescents: 1994-2010. Pediatric Obesity.

[9] Bleich SN, Wolfson JA (2015). U.S. adults and child snacking patterns among sugar-sweetened beverage drinkers and non-drinkers. Prev Med.

[10] Piernas C, Ng SW, Popkin B (2013). Trends in purchases and intake of foods and beverages containing caloric and low-calorie sweeteners over the last decade in the United States. Pediatric Obesity.

[11] Poti JM, Popkin BM (2011). Trends in energy intake among US children by eating location and food source, 1977–2006. J Am Diet Assoc.

[12] Gordon-Larsen P (2014). Food availability/convenience and obesity. Adv Nutr (Bethesda, MD).

[13] Ohri-Vachaspati P, Isgor Z, Rimkus L, Powell LM, Barker DC, Chaloupka FJ (2015). Child-directed marketing inside and on the exterior of fast food restaurants. Am J Prev Med.

[14] Larson Nicole I., Story Mary T., Nelson Melissa C. (2009). Neighborhood Environments. American Journal of Preventive Medicine.

[15] Morland KB, Evenson KR (2009). Obesity prevalence and the local food environment. Health Place.

[16] Vinci DM, Philipp SF (2007). Perceived value in food selection when dining out: comparison of African Americans and euro-Americans. Percept Mot Skills.

[17] Dennisuk LA, Coutinho AJ, Suratkar S, Surkan PJ, Christiansen K, Riley M, Ja A, Sharma S, Gittelsohn J (2011). Food expenditures and food purchasing among low-income, urban, African-American youth. Am J Prev Med.

[18] Borradaile KE, Sherman S, Vander Veur SS, McCoy T, Sandoval B, Nachmani J, Karpyn A, Foster GD (2009). Snacking in children: the role of urban corner stores. Pediatrics.

[19] Economos CD, Folta SC, Goldberg J, Hudson D, Collins J, Baker Z, Lawson E, Nelson M (2009). A community-based restaurant initiative to increase availability of healthy menu options in Somerville, Massachusetts: shape up Somerville. Prev Chronic Dis.

[20] Goldberg JP, Collins JJ, Folta SC, McLarney MJ, Kozower C, Kuder J, Clark V, Economos CD (2009). Retooling food service for early elementary school students in Somerville, Massachusetts: the shape up Somerville experience. Prev Chronic Dis.

[21] Economos CD, Hyatt RR, Must A, Goldberg JP, Kuder J, Naumova EN, Collins JJ, Nelson ME (2013). Shape up Somerville two-year results: a community-based environmental change intervention sustains weight reduction in children. Prev Med.

[22] Folta SC, Kuder JF, Goldberg JP, Hyatt RR, Must A, Naumova EN, Nelson ME, Economos CD (2013). Changes in diet and physical activity resulting from the shape up Somerville community intervention. BMC Pediatr.

[23] Song H, Gittelsohn J, Kim M, Suratkar S, Sharma S, Anliker J (2009). A corner store intervention in a low-income urban community is associated with increased availability and sales of some healthy foods. Public Health Nutr.

[24] Waters E, de Silva-Sanigorski A, Hall BJ, Brown T, Campbell KJ, Gao Y, Armstrong R, Prosser L, Summerbell CD: Interventions for preventing obesity in children. Cochrane Database Syst Rev 2011(12):CD001871. 10.1002/14651858.CD001871.pub3 22161367

[25] Wang Y, Cai L, Wu Y, Wilson RF, Weston C, Fawole O, Bleich SN, Cheskin LJ, Showell NN, Lau BD (2015). What childhood obesity prevention programmes work? A systematic review and meta-analysis. Obes Rev.

[26] St-Onge M-P, Keller KL, Heymsfield SB (2003). Changes in childhood food consumption patterns: a cause for concern in light of increasing body weights. Am J Clin Nutr.

[27] Gittelsohn J, Steeves EA, Mui Y, Kharmats A, Hopkins L, Dennis D (2014). B'More healthy communities for kids: Design of a Multi-Level Intervention for obesity prevention for low-income African American children. BMC Public Health.

[28] Franco M, Diez Roux AV, Glass TA, Caballero B, Brancati FL (2008). Neighborhood characteristics and availability of healthy foods in Baltimore. Am J Prev Med.

[29] Schwendler T, Shipley C, Budd N, Trude A, Surkan PJ, Anderson Steeves E, Sato PM, Eckmann T, Loh H, Gittelsohn J. Development and implementation: B’more healthy communities for kids store and wholesaler intervention. Health Promot Pract. 2017;1524839917696716. 10.1177/1524839917696716 PMC5729580 28343413

[30] B'more Healthy Communities for Kids Introduction Training Video [https://www.youtube.com/channel/UC2TYlH237PSKL6HNZ2AeEmQ].

[31] Perepezko K, Tingey L, Sato P, Rastatter S, Ruggiero C, Gittelsohn J (2018). Partnering with carryouts: implementation of a food environment intervention targeting youth obesity. Health Educ Res.

[32] Trude ACB, Anderson Steeves E, Shipley C, Surkan PJ, Sato PM, Estep T, Clanton S, Lachenmayr L, Gittelsohn J (2018). A youth-leader program in Baltimore City recreation centers: lessons learned and applications. Health Promot Pract.

[33] Bandura A (2004). Health promotion by social cognitive means. Health Educ Behav.

[34] Sato PM, Steeves EA, Carnell S, Cheskin LJ, Trude AC, Shipley C, Mejía Ruiz MJ, Gittelsohn J (2016). A youth mentor-led nutritional intervention in urban recreation centers: a promising strategy for childhood obesity prevention in low-income neighborhoods. Health Educ Res.

[35] Loh IH, Schwendler T, Trude ACB, Anderson Steeves ET, Cheskin LJ, Lange S, Gittelsohn J (2018). Implementation of text-messaging and social media strategies in a multilevel childhood obesity prevention intervention: process evaluation results. Inquiry.

[36] Gittelsohn Joel, Mui Yeeli, Adam Atif, Lin Sen, Kharmats Anna, Igusa Takeru, Lee Bruce Y. (2015). Incorporating Systems Science Principles into the Development of Obesity Prevention Interventions: Principles, Benefits, and Challenges. Current Obesity Reports.

[37] Nam CS, Ross A, Ruggiero C, Ferguson M, Mui Y, Lee BY, Gittelsohn J: Process evaluation and lessons learned from engaging local policymakers in the B’More healthy communities for kids trial. Health Educ Behav 2018, 00(0):1090198118778323. 10.1177/1090198118778323 PMC6440198 29969930

[38] Dodson JL, Hsiao Y-C, Kasat-Shors M, Murray L, Nguyen NK, Richards AK, Gittelsohn J (2009). Formative research for a healthy diet intervention among inner-city adolescents: the importance of family, school and neighborhood environment. Ecol Food Nutr.

[39] Sonneville KR, Gortmaker SL (2008). Total energy intake, adolescent discretionary behaviors and the energy gap. Int J Obes.

[40] Sattler M, Hopkins L, Anderson Steeves E, Cristello A, McCloskey M, Gittelsohn J, Hurley K (2015). Characteristics of youth food preparation in low-income, African American homes: associations with healthy eating index scores. Ecol Food Nutr.

[41] Trude ACB, Kharmats AY, Hurley KM, Anderson Steeves E, Talegawkar SA, Gittelsohn J (2016). Household, psychosocial, and individual-level factors associated with fruit, vegetable, and fiber intake among low-income urban African American youth. BMC Public Health.

[42] Gittelsohn J, Kim EM, He S, Pardilla M (2013). A food store-based environmental intervention is associated with reduced BMI and improved psychosocial factors and food-related behaviors on the Navajo nation. J Nutr.

[43] Gittelsohn J, Ja A, Sharma S, Vastine AE, Caballero B, Ethelbah B (2006). Psychosocial determinants of food purchasing and preparation in American Indian households. J Nutr Educ Behav.

[44] Suratkar S, Gittelsohn J, Song H-J, Anliker JA, Sharma S, Mattingly M (2010). Food insecurity is associated with food-related psychosocial factors and behaviors among low-income African American adults in Baltimore City. J Hunger Environ Nutr.

[45] Shin A, Surkan PJ, Coutinho AJ, Suratkar SR, Campbell RK, Rowan M, Sharma S, Dennisuk LA, Karlsen M, Gass A (2015). Impact of Baltimore healthy eating zones: an environmental intervention to improve diet among African American youth. Health Educ Behav.

[46] Lent MR, Vander Veur S, Mallya G, McCoy TA, Sanders TA, Colby L, Rauchut Tewksbury C, Lawman HG, Sandoval B, Sherman S (2014). Corner store purchases made by adults, adolescents and children: items, nutritional characteristics and amount spent. Public Health Nutr.

[47] Cullen KW, Watson K, Zakeri I (2008). Relative reliability and validity of the block kids questionnaire among youth aged 10 to 17 years. J Am Diet Assoc.

[48] Smith Chery, Fila Stefanie (2006). Comparison of the Kid's Block Food Frequency Questionnaire to the 24-hour recall in urban Native American youth. American Journal of Human Biology.

[49] Vedovato GM, Surkan PJ, Jones-Smith J, Steeves EA, Han E, Trude ACB, Kharmats AY, Gittelsohn J (2016). Food insecurity, overweight and obesity among low-income African-American families in Baltimore City: associations with food-related perceptions. Public Health Nutr.

[50] Gittelsohn J, Song H-J, Suratkar S, Kumar MB, Henry EG, Sharma S, Mattingly M, Anliker JA (2010). An urban food store intervention positively affects food-related psychosocial variables and food behaviors. Health Educ Behav.

[51] Willett WC, Howe GR, Kushi LH (1997). Adjustment for total energy intake in epidemiologic studies. Am J Clin Nutr.

[52] Fitzmaurice GM, Laird NM, Ware JH (2012). Applied longitudinal analysis.

[53] Ruggiero CF, Poirier L, Trude ACB, Yang T, Schwendler T, Gunen B, Loh IH, Perepezko K, Nam CS, Sato P, et al. Implementation of B'More healthy communities for kids: process evaluation of a multi-level, multi-component obesity prevention intervention. Health Educ Res. 2018. 10.1093/her/cyy031 PMC6293311 30202959

[54] Gittelsohn J, Trude A, Poirier L, Ross A, Ruggiero C, Schwendler T, Anderson Steeves E (2017). The impact of a multi-level multi-component childhood obesity prevention trial on healthy food availability, sales, and purchasing in a low-income urban area. Int J Environ Res Publ Health.

[55] Slining MM, Mathias KC, Popkin BM (2013). Trends in food and beverage sources among US children and adolescents: 1989-2010. J Acad Nutr Diet.

[56] Hoelscher DM, Springer AE, Ranjit N, Perry CL, Evans AE, Stigler M, Kelder SH (2010). Reductions in child obesity among disadvantaged school children with community involvement: the Travis County CATCH trial. Obesity.

[57] Marcus C, Nyberg G, Nordenfelt A, Karpmyr M, Kowalski J, Ekelund U (2009). A 4-year, cluster-randomized, controlled childhood obesity prevention study: STOPP. Int J Obes.

[58] Gu X, Tucker KL (2016). Dietary quality of the US child and adolescent population: trends from 1999 to 2012 and associations with the use of federal nutrition assistance programs. Am J Clin Nutr.

[59] Bassett R, Chapman GE, Beagan BL (2008). Autonomy and control: the co-construction of adolescent food choice. Appetite.

[60] Taylor RW, McAuley KA, Barbezat W, Strong A, Williams SM, Mann JI (2007). APPLE project: 2-y findings of a community-based obesity prevention program in primary school age children. Am J Clin Nutr.

[61] Gentile DA, Welk G, Eisenmann JC, Reimer RA, Walsh DA, Russell DW, Callahan R, Walsh M, Strickland S, Fritz K (2009). Evaluation of a multiple ecological level child obesity prevention program: switch what you do, view, and chew. BMC Med.

[62] Cohen JFW, Kraak VI, Choumenkovitch SF, Hyatt RR, Economos CD (2014). The CHANGE study: a healthy-lifestyles intervention to improve rural children's diet quality. J Acad Nutr Diet.

[63] Kit BK, Fakhouri TH, Park S, Nielsen SJ, Ogden CL (2013). Trends in sugar-sweetened beverage consumption among youth and adults in the United States: 1999-2010. Am J Clin Nutr.

[64] Epstein LH, Handley EA, Dearing KK, Cho DD, Roemmich JN, Paluch RA, Raja S, Pak Y, Spring B (2006). Purchases of food in youth: influence of Price and income. Psychol Sci.

[65] Trude ACB, Kharmats AY, Jones-Smith JC, Gittelsohn J (2018). Exposure to a multi-level multi-component childhood obesity prevention community-randomized controlled trial: patterns, determinants, and implications. Trials.

[66] Heerman WJ, JaKa MM, Berge JM, Trapl ES, Sommer EC, Samuels LR, Jackson N, Haapala JL, Kunin-Batson AS, Olson-Bullis BA (2017). The dose of behavioral interventions to prevent and treat childhood obesity: a systematic review and meta-regression. Int J Behav Nutr Phys Act.

