# Supplementary material for: A multilevel, multicomponent childhood obesity prevention group-randomized controlled trial improves healthier food purchasing and reduces sweet-snack consumption among low-income African-American youth
Source: Nutr J. 2018 Oct 29;17:96. doi: 10.1186/s12937-018-0406-2 (PMC6206663; doi:10.1186/s12937-018-0406-2)

Logo

Shelf Talkers

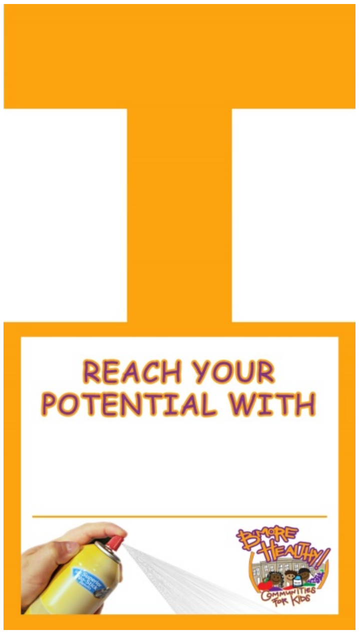

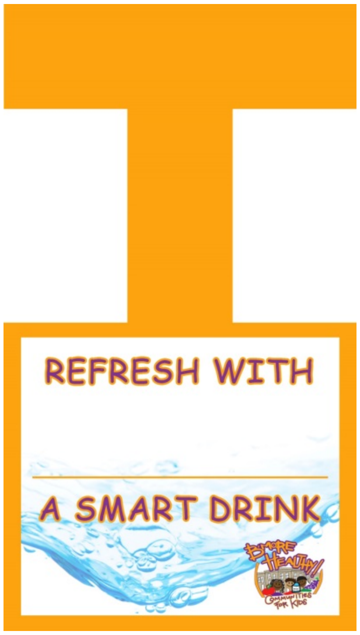

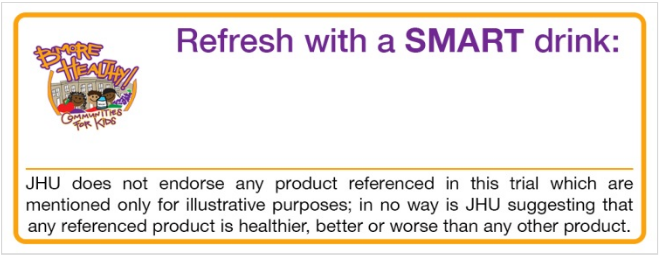

Posters

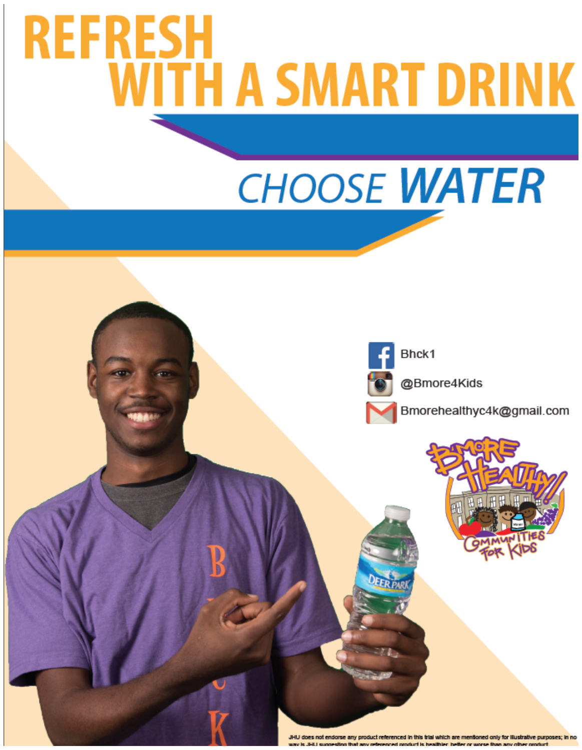

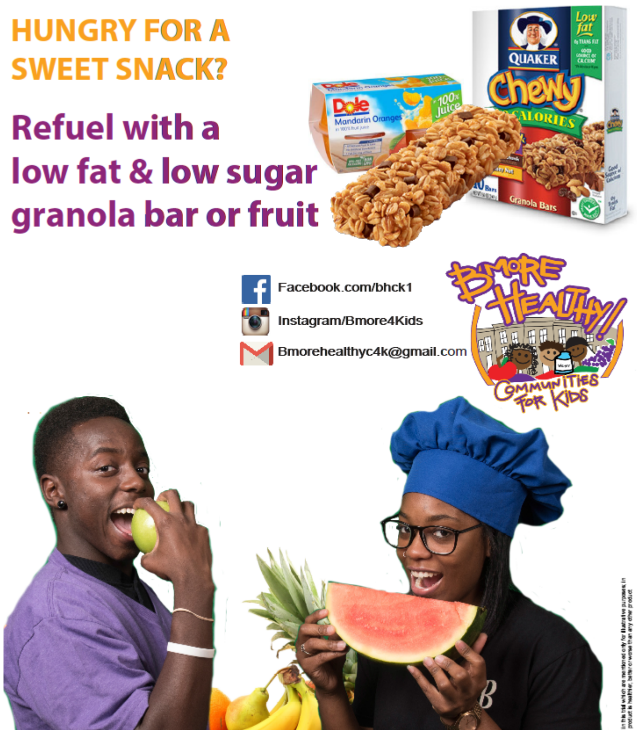

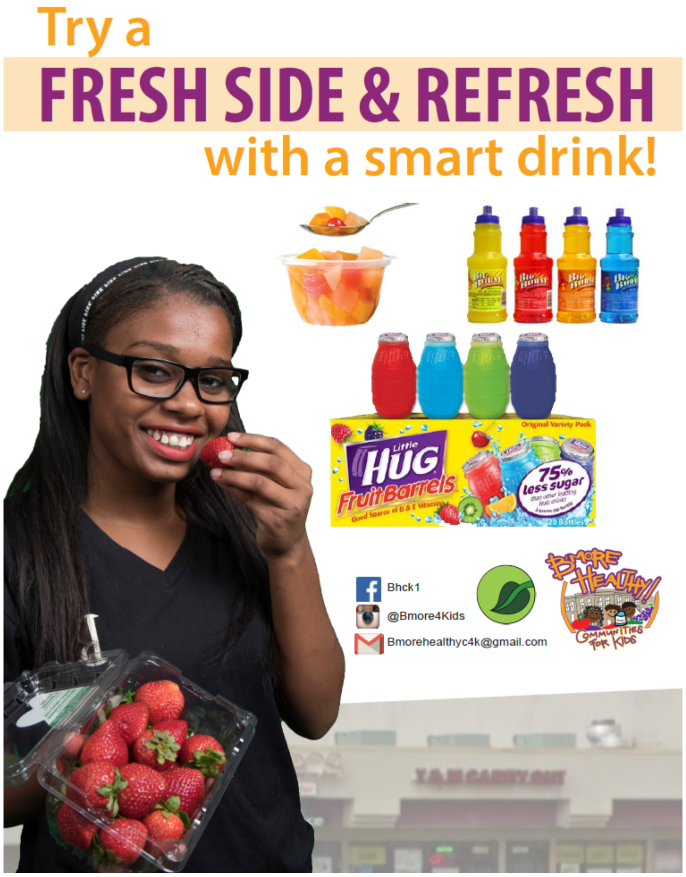
Try a
FRESH SIDE & REFRESH
with a smart drink!
Little HUG Fruit Barrels
75% less sugar
Bhck1
@Bmore4Kids
Bmorehealthyc4k@gmail.com
Bmore Healthy Communities for Kids

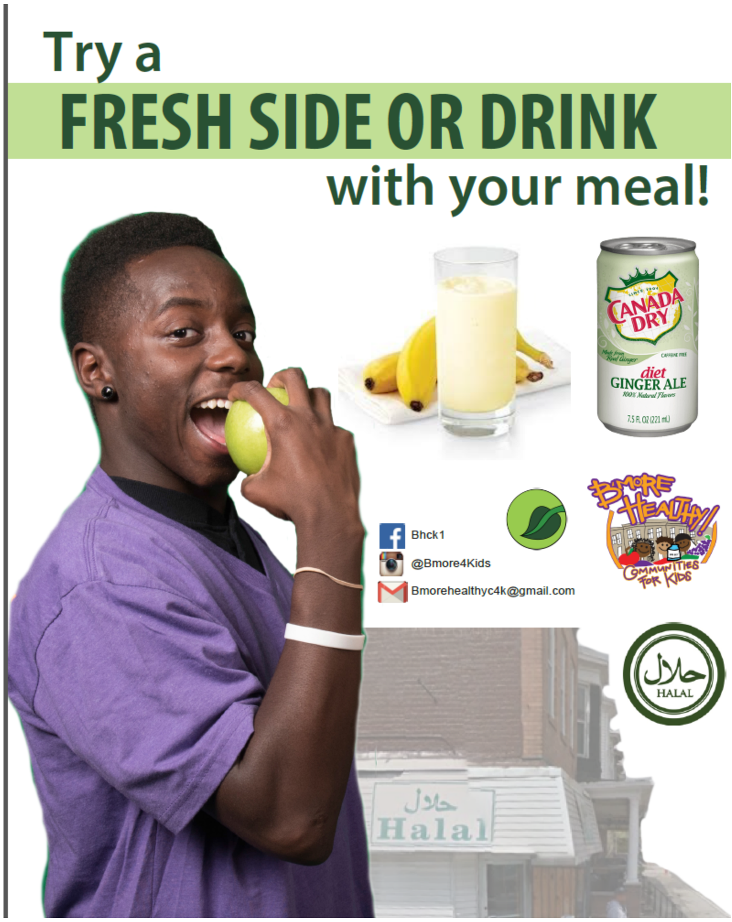
Try a
FRESH SIDE OR DRINK
with your meal!
CANADA DRY
diet GINGER ALE
Bhck1
@Bmore4Kids
Bmorehealthyc4k@gmail.com
Bmore Healthy Communities for Kids
HALAL
Halal

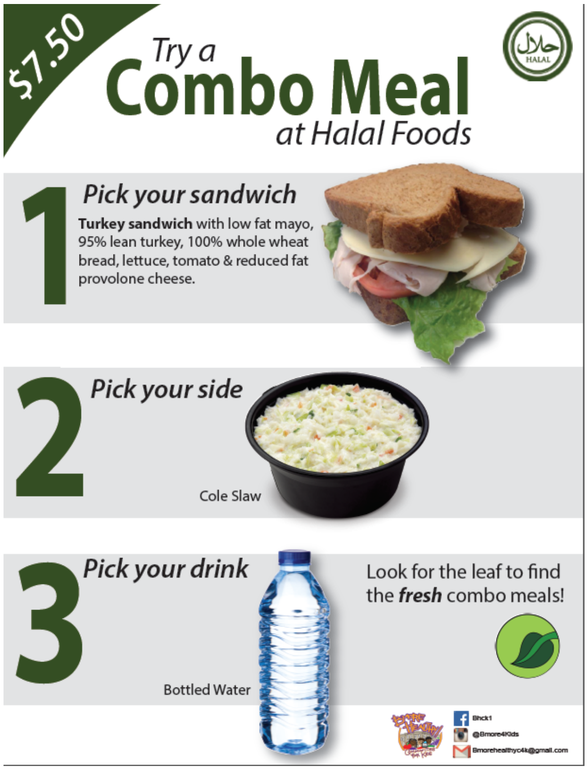
$7.50
Try a
Combo Meal
at Halal Foods
HALAL
1 Pick your sandwich
Turkey sandwich with low fat mayo, 95% lean turkey, 100% whole wheat bread, lettuce, tomato & reduced fat provolone cheese.
2 Pick your side
Cole Slaw
3 Pick your drink
Bottled Water
Look for the leaf to find the fresh combo meals!

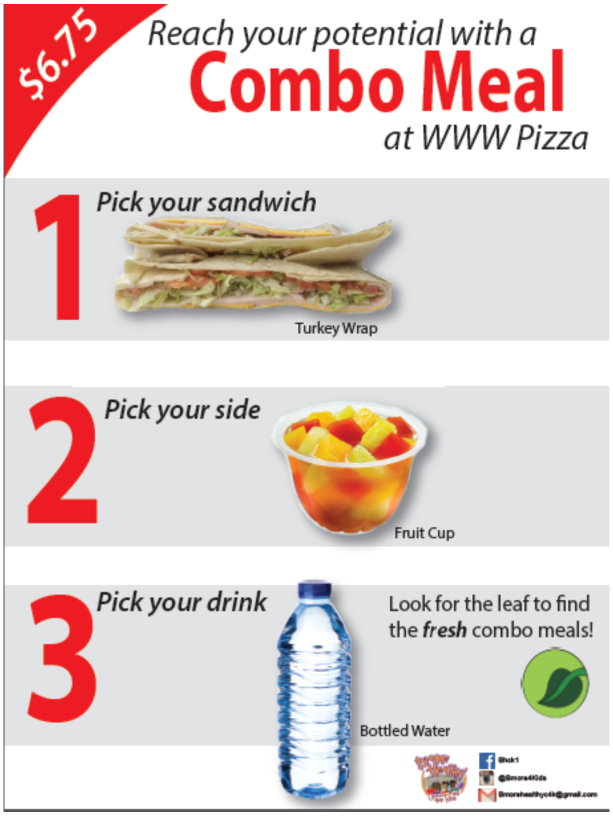
$6.75
Reach your potential with a
Combo Meal
at WWW Pizza
1 Pick your sandwich
Turkey Wrap
2 Pick your side
Fruit Cup
3 Pick your drink
Bottled Water
Look for the leaf to find the fresh combo meals!

Handouts

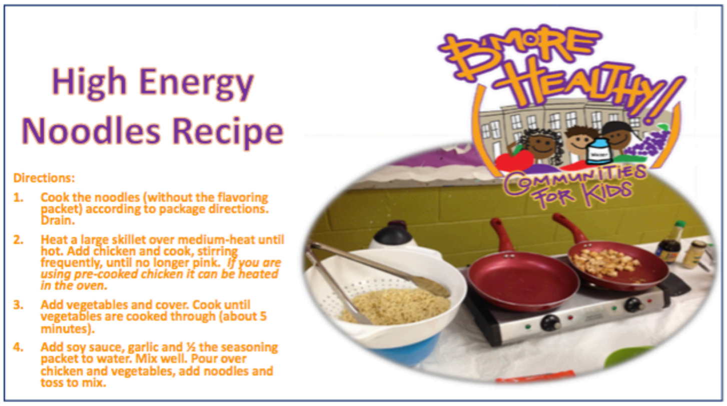

High Energy Noodles Recipe
Directions:
1. Cook the noodles (without the flavoring packet) according to package directions. Drain.
2. Heat a large skillet over medium-heat until hot. Add chicken and cook, stirring frequently, until no longer pink. If you are using pre-cooked chicken it can be heated in the oven.
3. Add vegetables and cover. Cook until vegetables are cooked through (about 5 minutes).
4. Add soy sauce, garlic and ½ the seasoning packet to water. Mix well. Pour over chicken and vegetables, add noodles and toss to mix.
B'MORE HEALTHY!
COMMUNITIES FOR KIDS

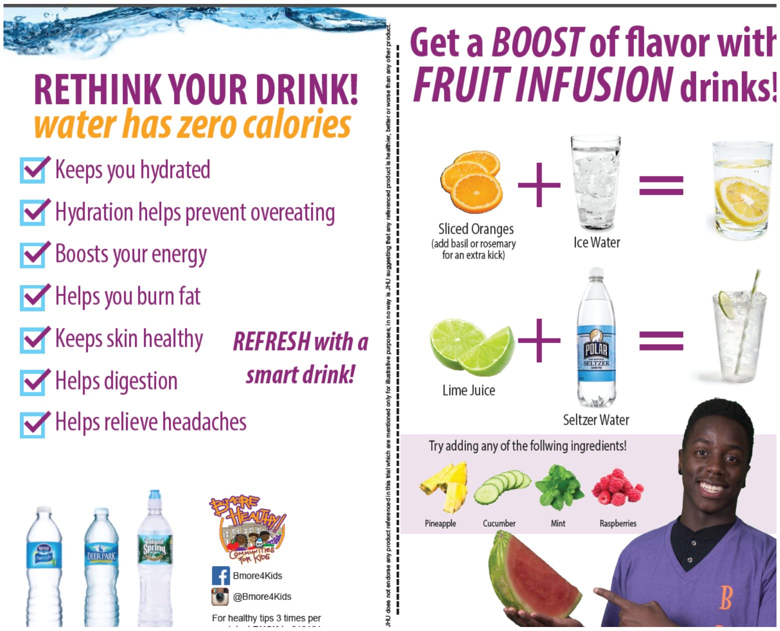

RETHINK YOUR DRINK!
water has zero calories
Keeps you hydrated
Hydration helps prevent overeating
Boosts your energy
Helps you burn fat
Keeps skin healthy
Helps digestion
Helps relieve headaches
REFRESH with a smart drink!
Bmore4Kids
@Bmore4Kids
For healthy tips 3 times per
Get a BOOST of flavor with FRUIT INFUSION drinks!
Sliced Oranges
(add basil or rosemary for an extra kick)
Ice Water
Lime Juice
Seltzer Water
Try adding any of the follwing ingredients!
Pineapple
Cucumber
Mint
Raspberries

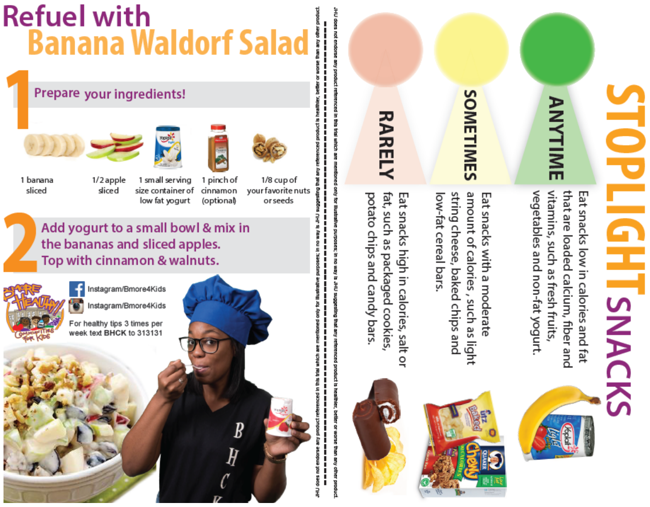

Refuel with Banana Waldorf Salad
1 Prepare your ingredients!
1 banana sliced
1/2 apple sliced
1 small serving size container of low fat yogurt
1 pinch of cinnamon (optional)
1/8 cup of your favorite nuts or seeds
2 Add yogurt to a small bowl & mix in the bananas and sliced apples. Top with cinnamon & walnuts.
Instagram/Bmore4Kids
Instagram/Bmore4Kids
For healthy tips 3 times per week text BHCK to 313131
STOPLIGHT SNACKS
ANYTIME
Eat snacks low in calories and fat that are loaded calcium, fiber and vitamins, such as fresh fruits, vegetables and non-fat yogurt.
SOMETIMES
Eat snacks with a moderate amount of calories, such as light string cheese, baked chips and low-fat cereal bars.
RARELY
Eat snacks high in calories, salt or fat, such as packaged cookies, potato chips and candy bars.

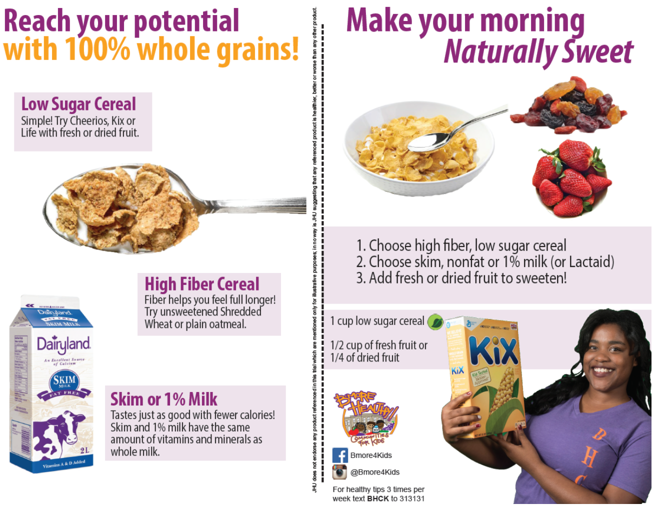

Reach your potential with 100% whole grains!
Low Sugar Cereal
Simple! Try Cheerios, Kix or Life with fresh or dried fruit.
High Fiber Cereal
Fiber helps you feel full longer! Try unsweetened Shredded Wheat or plain oatmeal.
Skim or 1% Milk
Tastes just as good with fewer calories! Skim and 1% milk have the same amount of vitamins and minerals as whole milk.
Make your morning Naturally Sweet
1. Choose high fiber, low sugar cereal
2. Choose skim, nonfat or 1% milk (or Lactaid)
3. Add fresh or dried fruit to sweeten!
1 cup low sugar cereal
1/2 cup of fresh fruit or 1/4 of dried fruit
Bmore4Kids
@Bmore4Kids
For healthy tips 3 times per week text BHCK to 313131

Giveaways

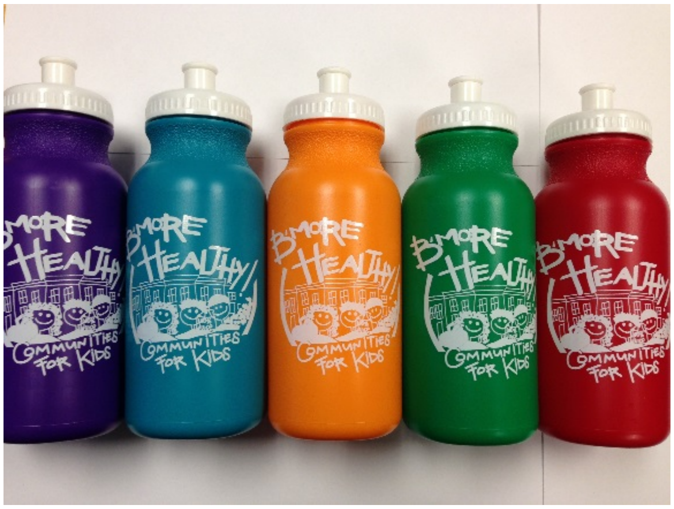
B'MORE HEALTHY!
COMMUNITIES FOR KIDS

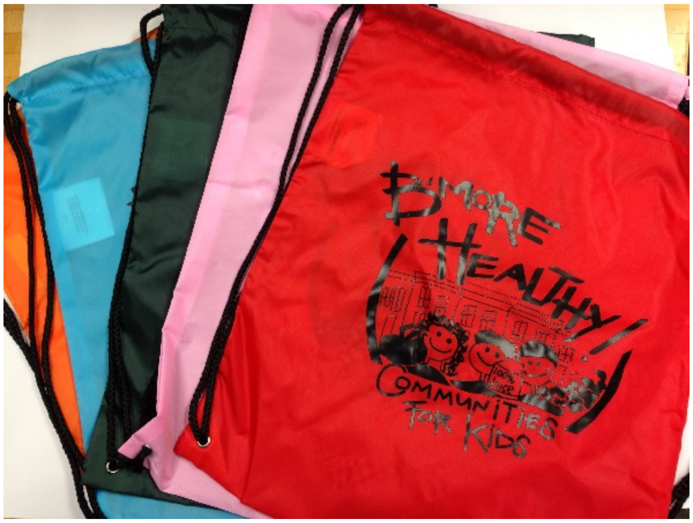
B'MORE HEALTHY!
COMMUNITIES FOR KIDS

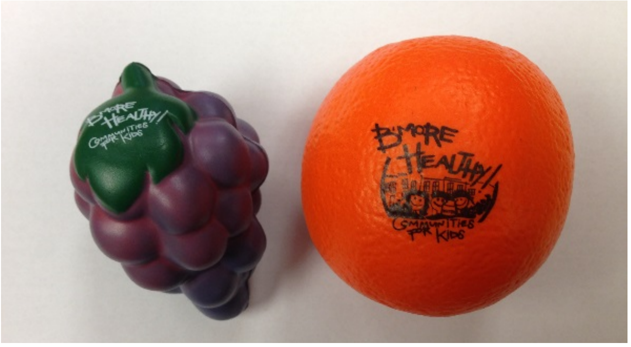
B'MORE HEALTHY!
COMMUNITIES FOR KIDS

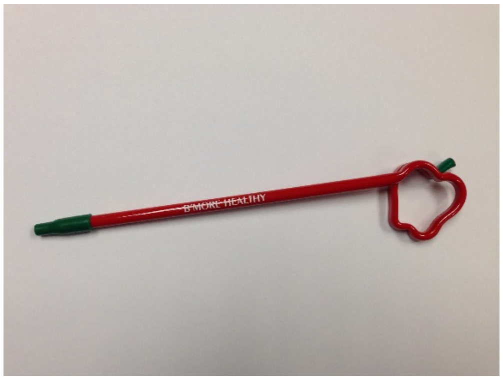
B'MORE HEALTHY

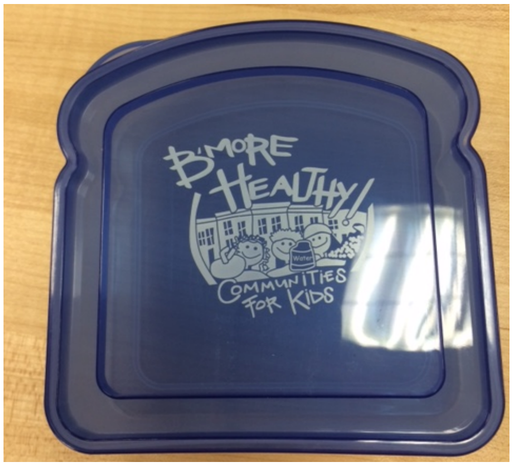
B'MORE HEALTHY!
COMMUNITIES FOR KIDS

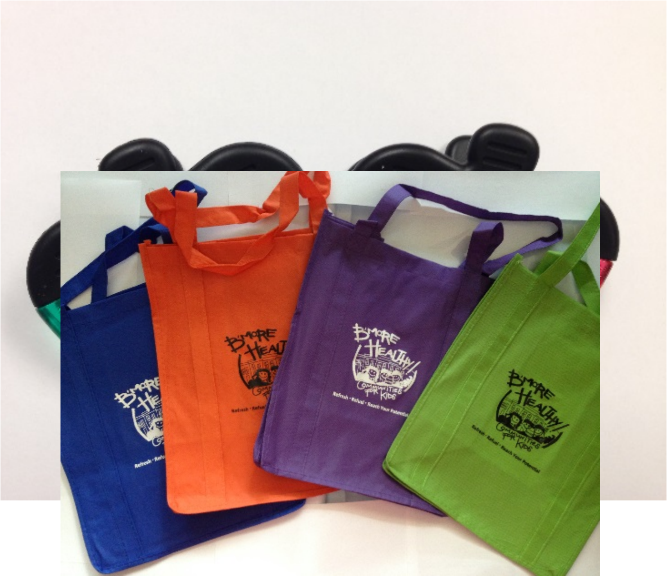

Educational Activities

Rethink your drink #1

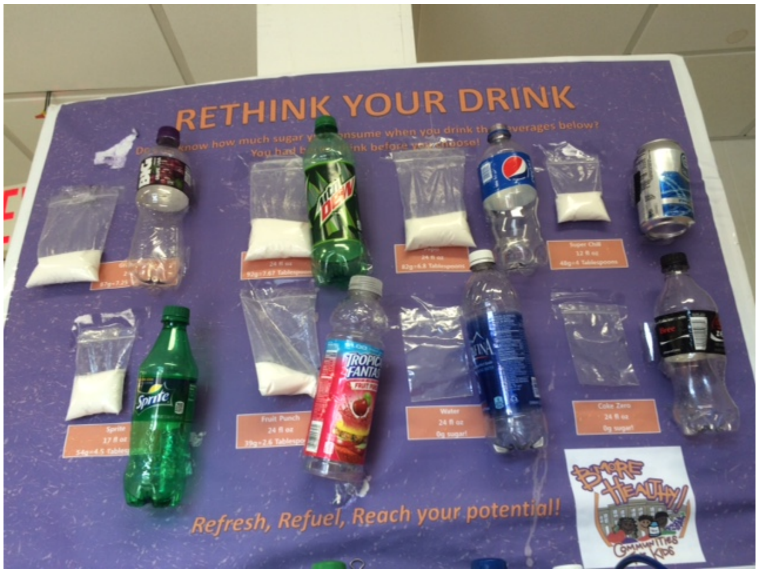

Do you look at the nutrition label?

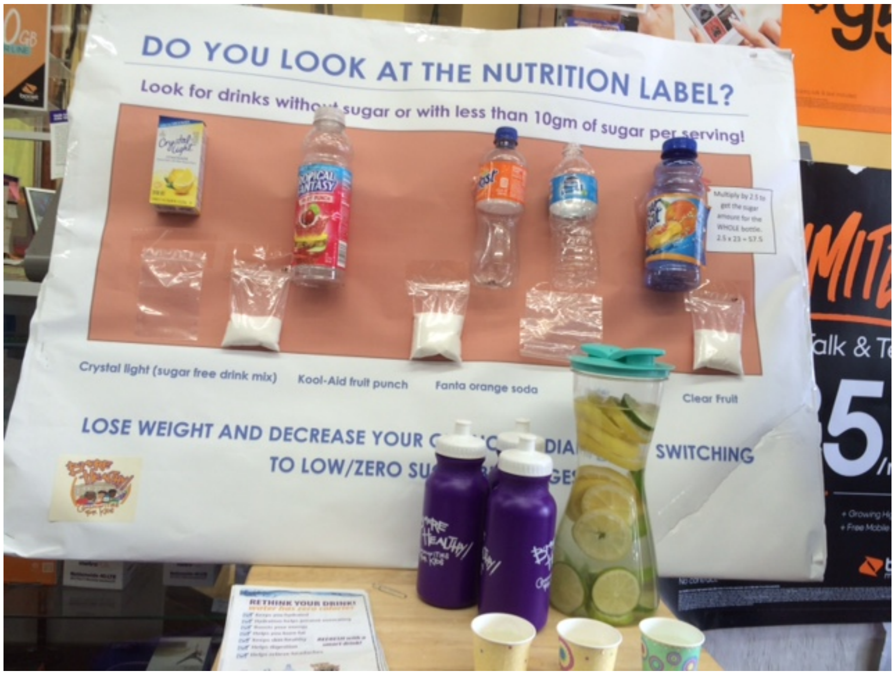

Have a craving for something sweet?

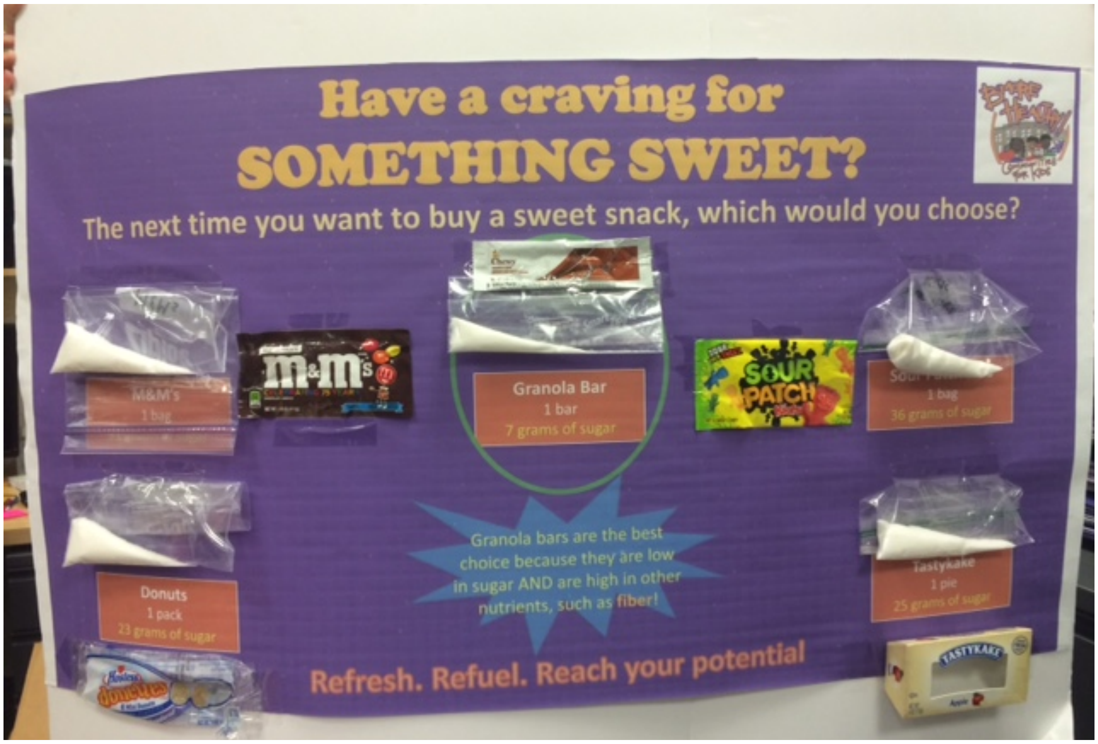

Carryout Menus

HALAL FOODS
4202 Park Heights Ave. (443) 449-7192

Bhck1
@Bmore4Kids

SALADS
*Dressing included on the side

Garden Salad $___.___

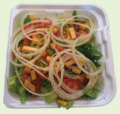

Chicken Salad $___.___

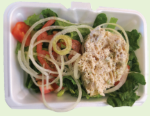

Salmon Salad $___.___
Tuna Salad $___.___

BURGERS & FRANKS
Turkey burger $___.___
Cheese burger $___.___
Beef burger $___.___
Beef frank $___.___

SEAFOOD
Fish and fries $___.___
Fish bites $___.___
Crab cake $___.___
Salmon cake $___.___
Crab & salmon cake $___.___
Shrimp crab cake $___.___

CHICKEN
3 wings/fries $___.___
5 wings/fries $___.___
7 wings/fries $___.___
3 wings $___.___
5 wings $___.___
7 wings $___.___

FISH
Shrimp fish special $___.___
Fish omelet $___.___
Fish sub $___.___
Jumbo fish sandwich $___.___
Fish sandwich $___.___
Fish special $___.___

SPECIALS
Ballers Subs/Wraps $___.___
Crabmeat, fish, shrimp, cheese & onions
Choice of yellow rice or fries for $___.___
Mushrooms or green peppers for $___.___

The "88" $___.___
Chicken, fish, burger or steak & 4 toppings
Add shrimp or crabmeat for $___.___

BREAKFAST ITEMS
Waffles $___.___
Comes with meat & eggs
French Toast $___.___
Comes with meat & eggs
Pancakes $___.___
Comes with meat & eggs
Breakfast platter $___.___
Home fries or grits w/ meat & eggs
Sausage sandwich $___.___
Chicken, beef or turkey sausage w/egg
Breakfast platter $___.___
Turkey or beef bacon with egg
Omelets $___.___

Sorry, no credit cards accepted.

SIDE ORDERS
Cole slaw $___.___
Onion rings $___.___
French fries $___.___
Large fries $___.___
Chili/beef franks $___.___
Hot Dog $___.___
Chili Dog $___.___
Potato salad $___.___
Macaroni salad $___.___

SUBS
*All subs come with cheese
Mushroom cheese steak $___.___
Pepper cheese steak $___.___
Shrimp cheese steak $___.___
Cheese steak omelet $___.___
Steak $___.___
Shrimp chop chicken $___.___
Cheese chop chicken $___.___
Turkey cold cut $___.___

Look for the leaf for a fresh & delicious choice!

Recreation Center Activities

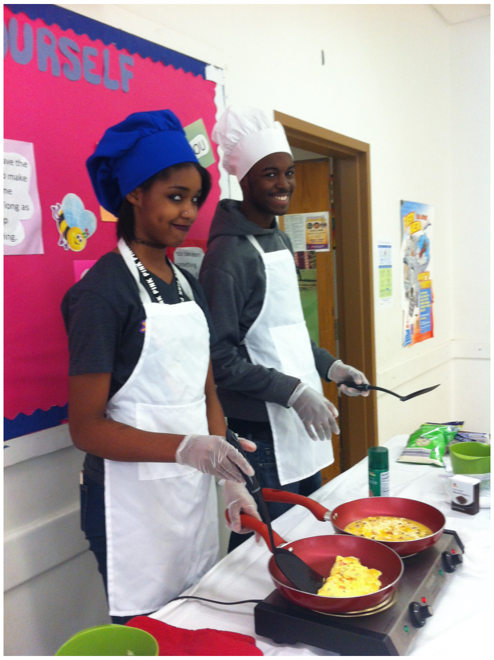

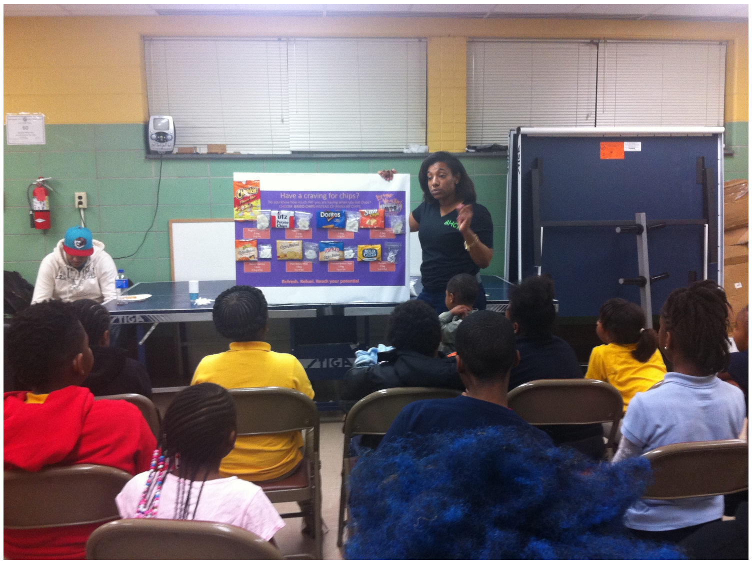

Social Media

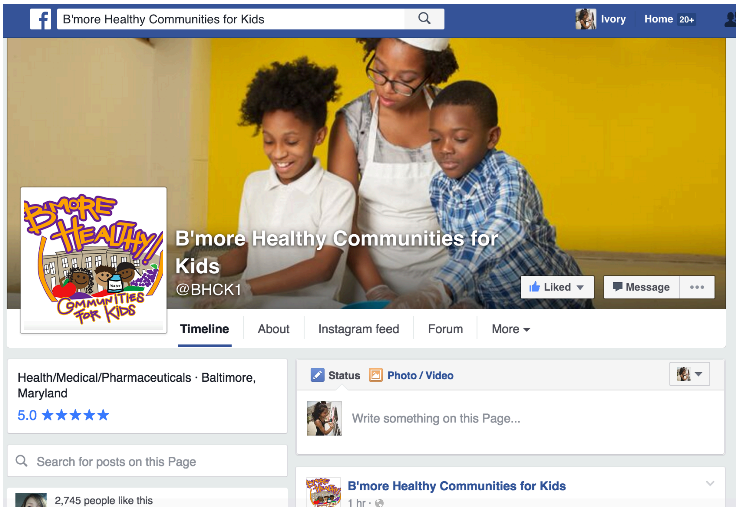

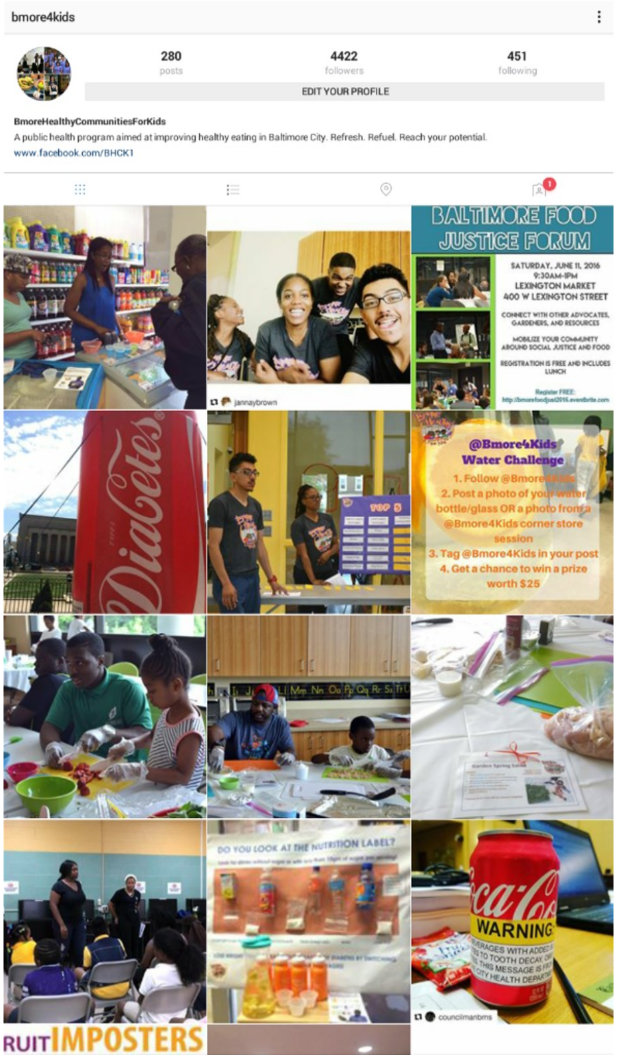

B'more Healthy Communities for Kids

Published by Ivory Loh [?] · June 11 at 7:00am ·

#FunFactFridayBHCK Did you know...that Raisin Bran has more #fiber than Corn Flakes and Kix? Corn Flakes and Kix are great low sugar cereal options but Raisin Bran wins in fiber! Refuel with these cereal in the morning! #RefuelBHCK

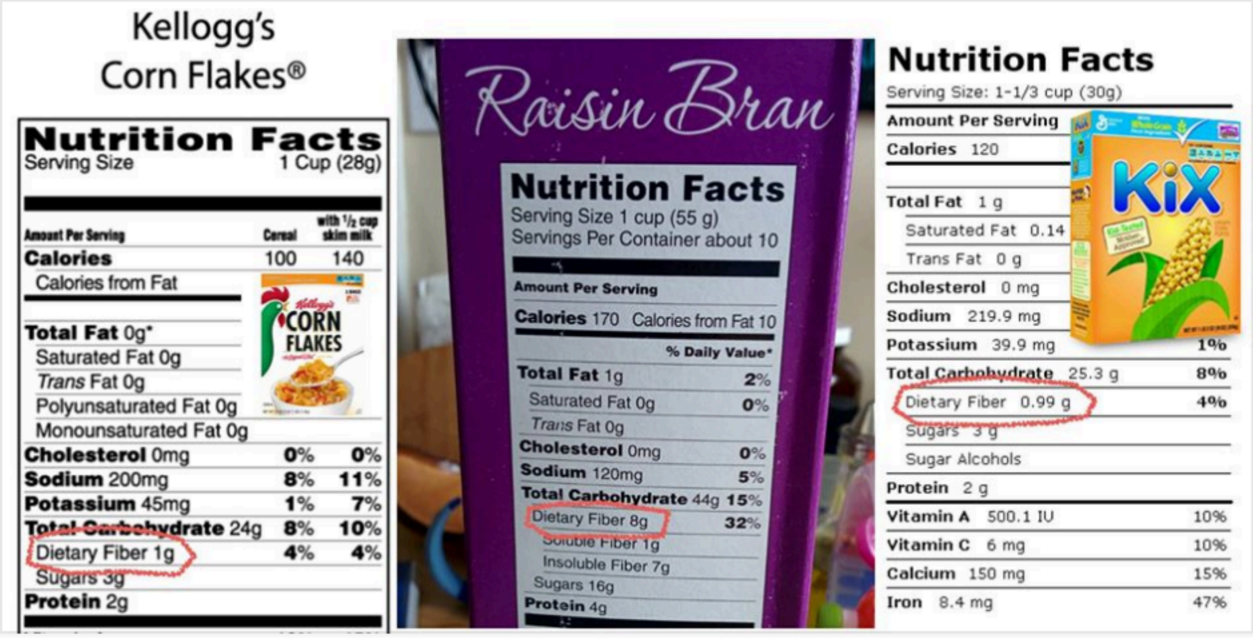

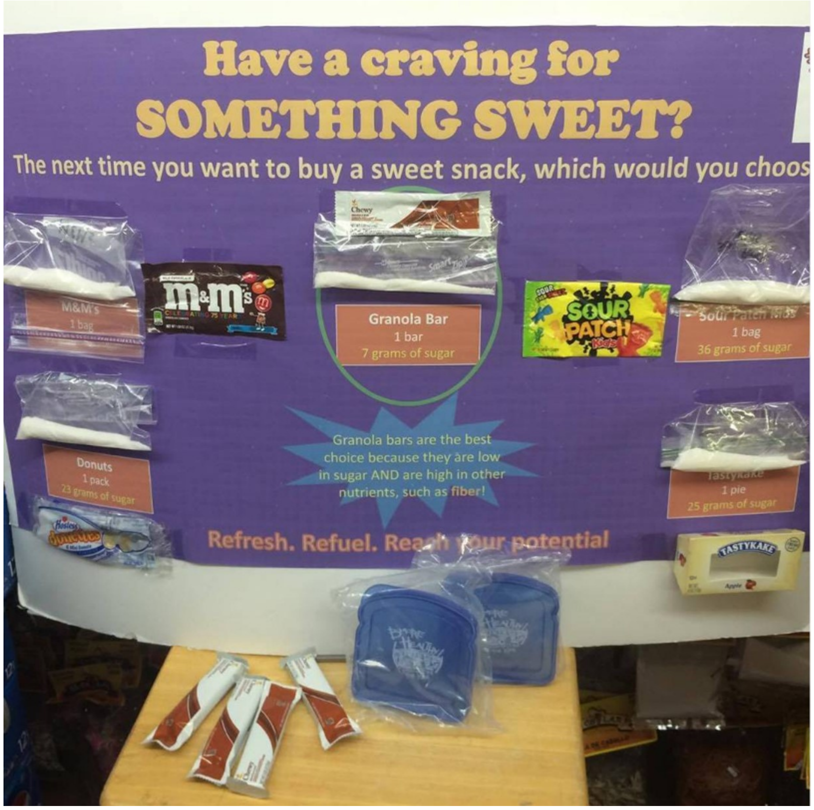

bmore4kids
Baltimore, Maryland

74 likes 4d

bmore4kids #Bmore4Kids is promoting chewy granola bars at #Baltimore corner stores this week! #DidYouKnow that chewy granola bars are a healthier alternative to candy and snack cakes because they are lower in sugar and higher in fiber? Check out our Facebook page (link in bio) to see which stores we are visiting, and stop by to grab a free granola bar!

#RefuelBHCK #SnackSmartBHCK #community #LowSugar #HealthyEats #HealthyKids #obesity #love #food #photooftheday #Maryland

leslieangel 💫✨

Add a comment...

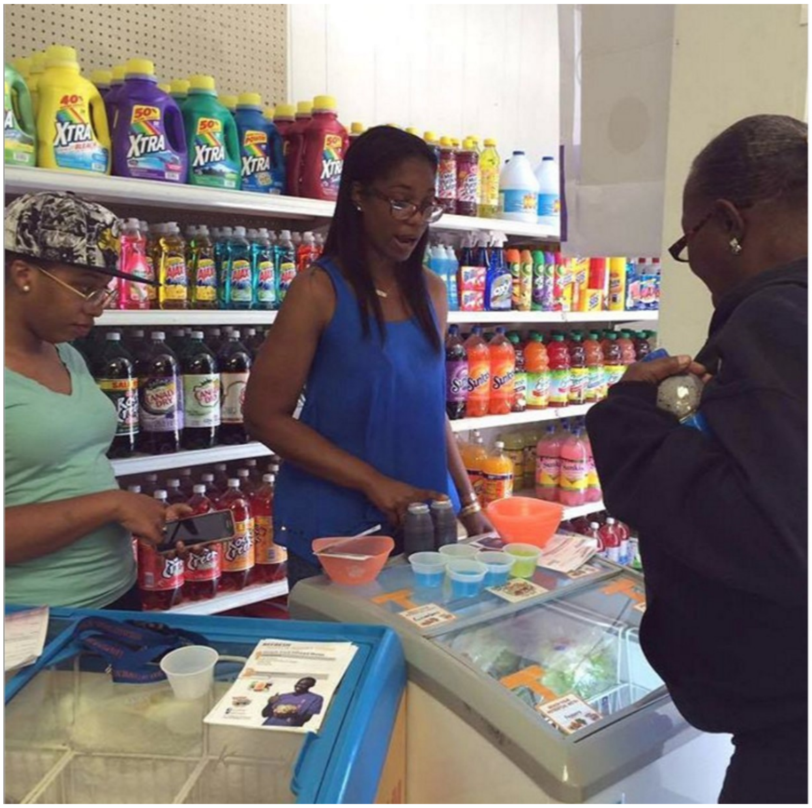

bmore4kids
Family Food Market

81 likes 4w

bmore4kids #DidYouKnow Hugs fruits drinks contain less sugar than soda? #Bmore4Kids Youth Leaders were at Family Food Market yesterday! They educate customers coming into corner stores and introduce them to low sugar drinks such as Hugs to help them make healthier choices.

#RefreshBHCK #DrinkSmartBHCK #CornerStore #youth #leaders #Baltimore #nutrition #education #obesity #FreshFood #GreenEats #LowSugar

Add a comment...

Mailing (wave 2 only)

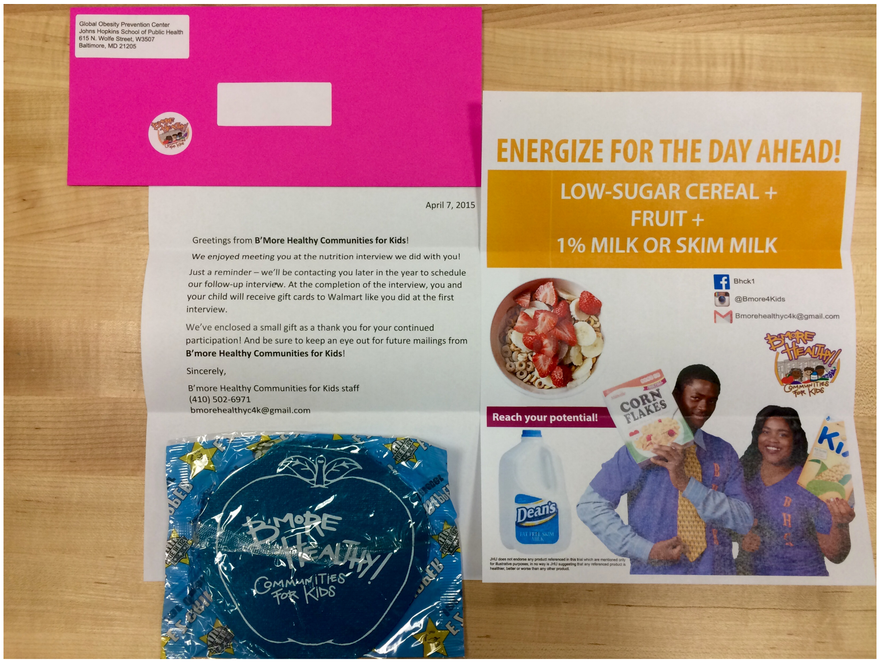

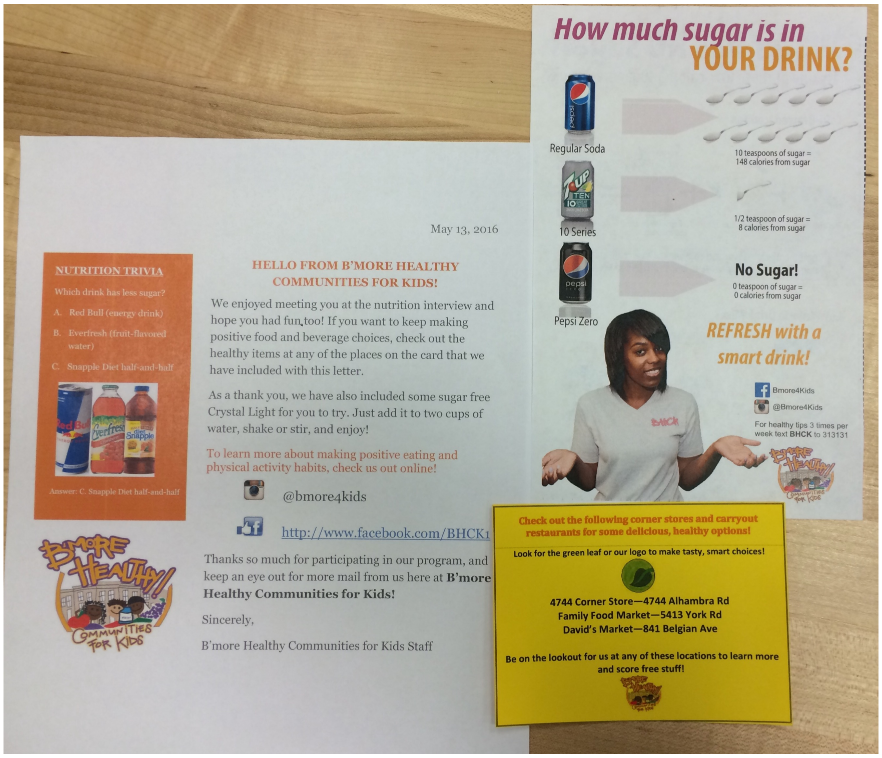

Supplement: Supplementary file 2 — This file contains pictures of communication and intervention materials used during the implementation of the B’more Healthy Communities for Kids intervention. (PDF 22812 kb) [file 12937_2018_406_MOESM2_ESM.pdf]
